# Inherited deletion of 9p22.3‐p24.3 and duplication of 18p11.31‐p11.32 associated with neurodevelopmental delay: Phenotypic matching of involved genes

**DOI:** 10.1111/jcmm.17662

**Published:** 2023-01-24

**Authors:** Naser Ajami, Mohammad Amin Kerachian, Mehran Beiraghi Toosi, Farah Ashrafzadeh, Susan Hosseini, Peter N. Robinson, Mohammad Reza Abbaszadegan

**Affiliations:** ^1^ Department of Medical Genetics and Molecular Medicine, Faculty of Medicine Mashhad University of Medical Sciences Mashhad Iran; ^2^ Medical Genetics Research Center Mashhad University of Medical Sciences Mashhad Iran; ^3^ Department of Pediatric Neurology, School of Medicine Mashhad University of Medical Sciences Mashhad Iran; ^4^ Neuroscience Research Center Mashhad University of Medical Sciences Mashhad Iran; ^5^ Pardis Pathobiology and Genetics Laboratory Mashhad Iran; ^6^ The Jackson Laboratory for Genomic Medicine Farmington Connecticut USA; ^7^ Immunology Research Center Mashhad University of Medical Sciences Mashhad Iran

**Keywords:** 9p deletion syndrome, DECIPHER, duplication of 18p, neurodevelopmental delay/intellectual disability, PhenogramViz

## Abstract

We describe a 3.5‐year‐old Iranian female child and her affected 10‐month‐old brother with a maternally inherited derivative chromosome 9 [der(9)]. The postnatally detected rearrangement was finely characterized by aCGH analysis, which revealed a 15.056 Mb deletion of 9p22.3‐p24.3p22.3 encompassing 14 OMIM morbid genes such as *DOCK8*, *KANK1*, *DMRT1* and *SMARCA2*, and a gain of 3.309 Mb on 18p11.31‐p11.32 encompassing *USP14*, *THOC1*, *COLEC12*, *SMCHD1* and *LPIN2*. We aligned the genes affected by detected CNVs to clinical and functional phenotypic features using PhenogramViz. In this regard, the patient's phenotype and CNVs data were entered into PhenogramViz. For the 9p deletion CNV, 53 affected genes were identified and 17 of them were matched to 24 HPO terms describing the patient's phenotypes. Also, for CNV of 18p duplication, 22 affected genes were identified and six of them were matched to 13 phenotypes. Moreover, we used DECIPHER for in‐depth characterization of involved genes in detected CNVs and also comparison of patient phenotypes with 9p and 18p genomic imbalances. Based on our filtration strategy, in the 9p22.3‐p24.3 region, approximately 80 pathogenic/likely pathogenic/uncertain overlapping CNVs were in DECIPHER. The size of these CNVs ranged from 12.01 kb to 18.45 Mb and 52 CNVs were smaller than 1 Mb in size affecting 10 OMIM morbid genes. The 18p11.31‐p11.32 region overlapped 19 CNVs in the DECIPHER database with the size ranging from 23.42 kb to 1.82 Mb. These CNVs affect eight haploinsufficient genes.

## 1 INTRODUCTION

Neurodevelopmental disorders (NDDs) are a heterogeneous class of conditions that impact brain development with deficits in several domains of cognitive function that affect daily life. It has been estimated that at least 30% of NDDs are caused by genetic factors.^1^ NDDs include intellectual disability (ID), autism spectrum disorder, and attention‐deficit/hyperactivity disorder.^2^

New high‐throughput technologies, such as whole exome and whole genome sequencing, have revolutionized the diagnostic approach for NDDs, but there is still an ongoing debate on the choice of the optimal first‐tier diagnostic test.^3^ However, for the detection of copy‐number variants (CNVs), array comparative genomic hybridization (aCGH), with an average diagnostic yield between 15% and 20%, continues to be widely used as a first‐tier diagnostic test.^4^ CNVs are not only a prevalent source of genomic variation in the general population, but are also a major cause of NDDs and related disorders.^5^

Here, we present a 3.5‐year‐old Iranian female child and her affected 10‐month‐old brother affected with derivative chromosome 9, originating from a balanced translocation t(9;18) (p22;p11.31) present in the mother. Characterization of the postnatally detected rearrangement by aCGH showed partial 9p monosomy and partial 18p trisomy. In line with the objectives and scope of this study, we matched patient's phenotypes to the genes within the detected CNVs using PhenogramViz. Facilitating a phenotype‐guided interpretation of CNVs, this software is available as a plugin for CytoScape (V.3.8.2) and integrates data from different sources (including OMIM, MGI, ZFIN and Orphanet) with the selected HPO terms and then visualizes gene‐to‐phenotype associations as a 2D network of nodes and edges, which is called a phenogram.^6^, ^7^ Furthermore, we used DECIPHER for in‐depth characterization of genes that overlap the detected CNVs along with other relevant information such as gene/disease association information and predictive scores (e.g., pLI score) and also comparison of patient phenotypes with 9p and 18p genomic imbalances. For finding specific gene–phenotype associations, small CNVs affecting only one gene was our focus.^8^

Since the first report of partial monosomy 9p in 1973,^9^ over 100 cases have been documented.^10^ Unlike full or mosaic trisomy 18 or partial trisomy 18q, trisomy 18p has been rarely reported with only about 32 cases of trisomy 18p having been published to date.^11^ Reported cases involving a translocation between chromosomes 9 and 18 resulting in partial monosomy 9p and partial trisomy 18p are even rarer. Our review of published reports found only one such report.^12^

## 2 MATERIALS AND METHODS

### 2.1 Blood sampling and karyotyping

Blood samples were collected from all members of the studied family. Informed consents were obtained. The study was approved by the regional ethics committee at the Mashhad University of Medical Sciences, Mashhad, Iran. Chromosomal culture and karyotyping of both children and the parents were performed on routinely cultured peripheral blood lymphocytes. A total of 20 metaphase spreads were analysed in each case according to the GTG banding technique. The maximum banding resolution achieved was 500–600 bands.

### 2.2 Copy number variation analysis using aCGH

Whole genome oligo aCGH was performed using SurePrint G3 ISCA V2 8X60K whole genome oligo array version 2 and was analysed using Agilent Cytogenomic software v4. The array consists of 60,000 spots with overall median probe spacing of 60 kb and higher near to 500 targeted disease regions. The sample was hybridized against the male sample used as reference.

### 2.3 Bioinformatic analysis

For aligning the genes affected by detected CNVs to clinical and functional phenotypic features, we used PhenogramViz software. The Human Phenotype Ontology (HPO) terms intellectual disability, neurodevelopmental delay, cognitive impairment, delayed speech and language development, trigonocephaly, corpus callosum atrophy, low posterior hairline, highly arched eyebrow, wide intermammillary distance, wide nasal bridge, long philtrum, thin upper lip vermilion, open mouth, downturned corners of the mouth, cryptorchidism, and patient's brain MRI findings together with a list of two detected CNVs were used for phenotypic matching.

For finding similar CNVs, the DECIPHER database was searched separately for individuals who had deletions of chromosome 9p and duplication of 18p, which was overlapped with the deletion and duplication identified in our case. Our filtering strategy was based on: CNVs of the same size or smaller than the CNVs identified in this study that were entirely contained with the 9p22.3‐p24.3 (204193–15260686) and 18p11.31‐p11.32(148963–3458388); Pathogenic/likely pathogenic/uncertain CNVs; patients harbouring one genotype and having recorded phenotypes. We focused more on small CNVs affecting only one gene for finding specific gene–phenotype associations.

## 3 RESULTS

### 3.1 Clinical assessment

A 3.5‐year‐old Iranian female child (Patient 1) and his 10‐month‐old brother (Patient 2) both with dysmorphic features and congenital malformations were referred for chromosomal analysis and genetic counselling. She was the first child born to healthy, non‐consanguineous parents after a full‐term pregnancy and normal delivery. The mother was aged 27 years and the father 31 years at the time of the baby's birth. Obstetric history revealed a history of one spontaneous abortion occurring at 10 weeks of gestational age. It seemed that patient 2 has similar complaints, which was determined upon clinical investigation. The remaining family history was unremarkable.

**FIGURE 1:**
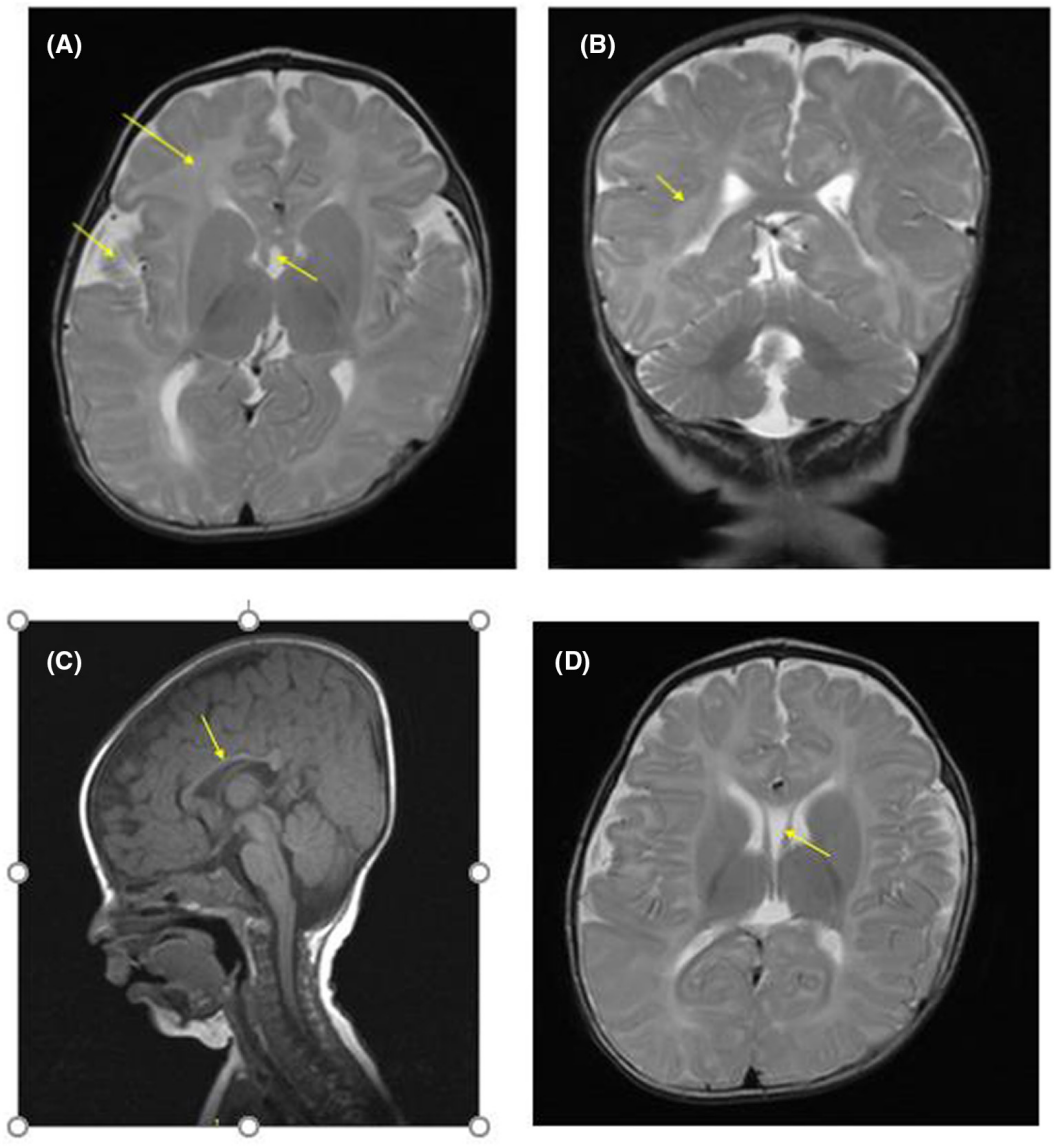
Brain MRI scan for patient 2 showing delayed myelination (A, uppermost arrow and B), enlarged sylvian cistern (A, left arrow), cavum septum pellucidum (A, right arrow and D), hypoplastic (thin) corpus callosum and trigonocephaly (C).

At clinical examination, patient 1's speech, gross motor and social milestones were prominently delayed. She had a trigonocephalic head, low posterior hairline, highly arched eyebrows, wide intermammillary distance, wide nasal bridge, long philtrum, thin upper lip vermilion, open mouth with downturned corners, mild micrognathia, hand flapping and repetitive movements, tapering fingers, and pes planus (Figure S1). She also had a small ventricular septal defect (VSD) as demonstrated by echocardiography. Her transferrin and iron‐binding capacity (TIBC) was high (500 μg/dl). Additional laboratory investigations, including brain and renal/urinary tract ultrasonography, blood urea, blood sugar, thyroid profile, creatinine, calcium, alkaline phosphatase, phosphorus, and serum electrolytes were all normal. Her brother had also relatively similar clinical manifestations along with cryptorchidism. Furthermore, his brain MRI showed a hypoplastic (thin) corpus callosum, cavum septum pellucidum, enlarged sylvian cistern, delayed myelination, and trigonocephaly (Figure 1).

### 3.2 Karyotyping and CNV analysis using aCGH

In all the metaphases from both children, a derivative chromosome 9 was detected, demonstrating a karyotype result of 46, XX, der(9),t(9;18)(p22;p11.31)mat and 46,XY,der(9),t(9;18)(p22;p11.31)mat. A normal karyotype was seen in the father. However, the mother's karyotype was 46, XX, t(9;18) (p22;p11.31) indicating that mother transmitted the detected abnormality to the children (Figure S2).

aCGH analysis of patient 1 showed the result of arr[GRCh37] 9p24.3p22.3 (204193–15260686)x1, arr[GRCh37] 18p11.32p11.31(148963–3458388)x3, indicating a 15.056 Mb deletion of 9p22.3‐p24.3 encompassing 122 genes including 43 genes listed in Online Mendelian Inheritance in Man (OMIM) and a gain of 3.309 Mb on 18p11.31‐p11.32 encompassing 42 genes including 16 genes listed in OMIM (Figure S3).

### 3.3 Phenotypic matching using PhenogramViz

For phenotypic matching, the patient's phenotype and CNV data were provided and entered to PhenogramViz. For the 9p deletion CNV, 53 affected genes were identified and 17 of them were matched to 24 HPO terms describing patient's phenotypes (Figure S4). Additionally, for the 18p duplication, 22 affected genes were identified and six of them were matched to 13 phenotypes (Figure S5).

### 3.4 Overlapped CNVs in the DECIPHER

We summarized the comparable cases of deleted CNVs of 9p22.3‐p24.3 (204193–15260686) and duplicated CNVs of 18p11.31‐p11.32 (148963–3458388) in the DECIPHER database. In the 9p22.3‐p24.3 region, approximately 80 pathogenic/likely pathogenic/uncertain CNVs were identified in DECIPHER based on our filtration strategy. The size ranged from 12.01 kb to 18.45 Mb and 52 CNVs were smaller than 1 Mb in size affecting 10 OMIM morbid genes, *NFIB*, *FREM1*, *DOCK8*, *GLDC*, *KANK1*, *KCNV2*, *GLIS3*, *TYRP1*, *JAK2* and *MPDZ* (Tables 1 and S1). Of note, 14 disease‐associated genes exist in the 9p22.3‐p24.3 region and four genes at 18p11.31‐p11.32, which are associated with diverse recessive and dominant Mendelian diseases (Tables 1 and S3). The remaining 28 CNVs with a size larger than 1 Mb harbour four other morbid genes *SMARCA2*, *VLDLR*, *SLC1A1* and *RIC1*.

**TABLE 1:** Morbid Genes in the region of 9p22.3‐p24.3 and 18p11.31‐p11.32

Cytoband	Gene	OMIM	pLI	%HI	G2P	Disease/s	PhenogramViz matched phenotype/s
9p24.3	*DOCK8*	611432	0	35.68	Biallelic	Hyper‐IgE recurrent infection syndrome (AR)	Intellectual disability, neurodevelopmental delay, global developmental delay, cognitive impairment, delayed speech and language development
	*KANK1*	607704	0	49.73	Imprinted (possible)	Cerebral palsy, spastic quadriplegic, 2 (U)	Hypoplasia of corpus callosum, enlarged sylvian cistern, cognitive impairment, intellectual disability, muscular hypotonia
	*SMARCA2*	600014	1	1.60	Monoallelic	Blepharophimosis‐impaired intellectual development syndrome (AD) Nicolaides–Baraitser syndrome (AD)	Intellectual disability, neurodevelopmental delay, global developmental delay, cognitive impairment, delayed speech and language development, motor delay, low posterior hairline, thick eyebrows, highly arched eyebrow, thin upper vermilion, long philtrum, open mouth, anteverted nostrils, wide nasal bridge, wide intermamillary distance, tapered finger, pes planus
9p24.2	*VLDLR*	192977	0	13.40	Biallelic	Cerebellar hypoplasia and mental retardation with or without quadrupedal locomotion 1 (AR)	Intellectual disability, neurodevelopmental delay, global developmental delay, cognitive impairment, delayed speech and language development, hypoplasia of corpus callosum, motor delay, pes planus
	*KCNV2*	607604	0	44.67	Biallelic	Retinal cone dystrophy 3B (AR)	
	*GLIS3*	610192	0	2.59	Biallelic	Hydrocephalus, congenital, 2, with or without brain or eye anomalies (AR)	
	*SLC1A1*	133550	0	31.17		Dicarboxylic aminoaciduria (AR) Schizophrenia susceptibility 18 (U)	Intellectual disability, cognitive impairment, hypoplasia of corpus callosum
9p24.1	*JAK2*	147796	0.65	0.82		Budd‐Chiari Syndrome; BDCHS (U) Erythrocytosis, Familial, 1; ECYT1 (U) Leukaemia, Acute Myeloid; AML (U) Myelofibrosis (U) Polycythaemia vera, somatic (U) Thrombocythemia 3 (AD, S)	Hypoplasia of corpus callosum, low posterior hairline, thick eyebrows, highly arched eyebrow, anteverted nostrils, wide nasal bridge, Elevated transferrin saturation
	*RIC1*	610354	0		Biallelic	CATIFA syndrome (AR)	
	*GLDC*	238300	0	30.92	Biallelic	Glycine encephalopathy (AR)	Intellectual disability, cognitive impairment, hypoplasia of corpus callosum, muscular hypotonia
9p23	*TYRP1*	115501	0	21.83	Biallelic	Albinism, oculocutaneous, type III (AR) Skin/hair/eye pigmentation, variation in, 11 (Melanesian blond hair) (U)	Hypoplasia of corpus callosum, low posterior hairline, highly arched eyebrow, thick eyebrow
	*MPDZ*	603785	0	33.44	Biallelic	Hydrocephalus, congenital, 2, with or without brain or eye anomalies (AR)	Enlarged sylvian cistern, intellectual disability, cognitive impairment
	*NFIB*		1	0.38	Monoallelic (possible)	Macrocephaly, acquired, with impaired intellectual development (AD)	Hypoplasia of corpus callosum, highly arched eyebrow, thick eyebrow
	*FREM1*	608944	0	26.80	Biallelic	Bifid nose with or without anorectal and renal anomalies (U) Manitoba oculotrichoanal syndrome (AR) Trigonocephaly 2 (AD)	Trigonocephaly, low posterior hairline, highly arched eyebrow, thick eyebrow, broad nasal beige, hypertelorism, anteverted nostrils, long philtrum, Tapered finger
18p11.32	*SMCHD1*	614982	1	27.68	Monoallelic	Bosma arhinia microphthalmia syndrome (AR) Facioscapulohumeral muscular dystrophy 2, digenic (Digenic dominant)	Low posterior hairline, highly arched eyebrow, thick eyebrow, pes planus
18p11.31	*LPIN2*	605519	0	38.0.45		Majeed syndrome (U)	
	*MYOM1*	603508	0	40.63		Hypertrophic Cardiomyopathy	
	*TGIF1*	602630	0.92	30.38	Monoallelic	Holoprosencephaly 4 (AD)	Trigonocephaly, hypoplasia of corpus callosum, cavum septum pellucidum, enlarged sylvian cistern, thick eyebrows, highly arched eyebrow, thin upper vermilion, long philtrum, anteverted nostrils, wide nasal bridge, hypertelorism

*Note*: pLI, The probability that a gene is intolerant to loss‐of‐function (LOF) mutations. Values range from 0 to 1 and genes with larger values (closer to one) are more intolerant of mutations. %HI, haploinsufficiency score, according to DECIPHER high ranks (e.g., 0%–10%) indicate that a gene is more likely to exhibit haploinsufficiency, low ranks (e.g., 90%–100%) indicate a gene is more likely to not exhibit haploinsufficiency. G2P, Gene2Phenotype is an online system designed to facilitate the development, validation, curation and distribution of large‐scale, evidence‐based datasets for use in diagnostic variant filtering (https://www.deciphergenomics.org/).

**FIGURE 2:**
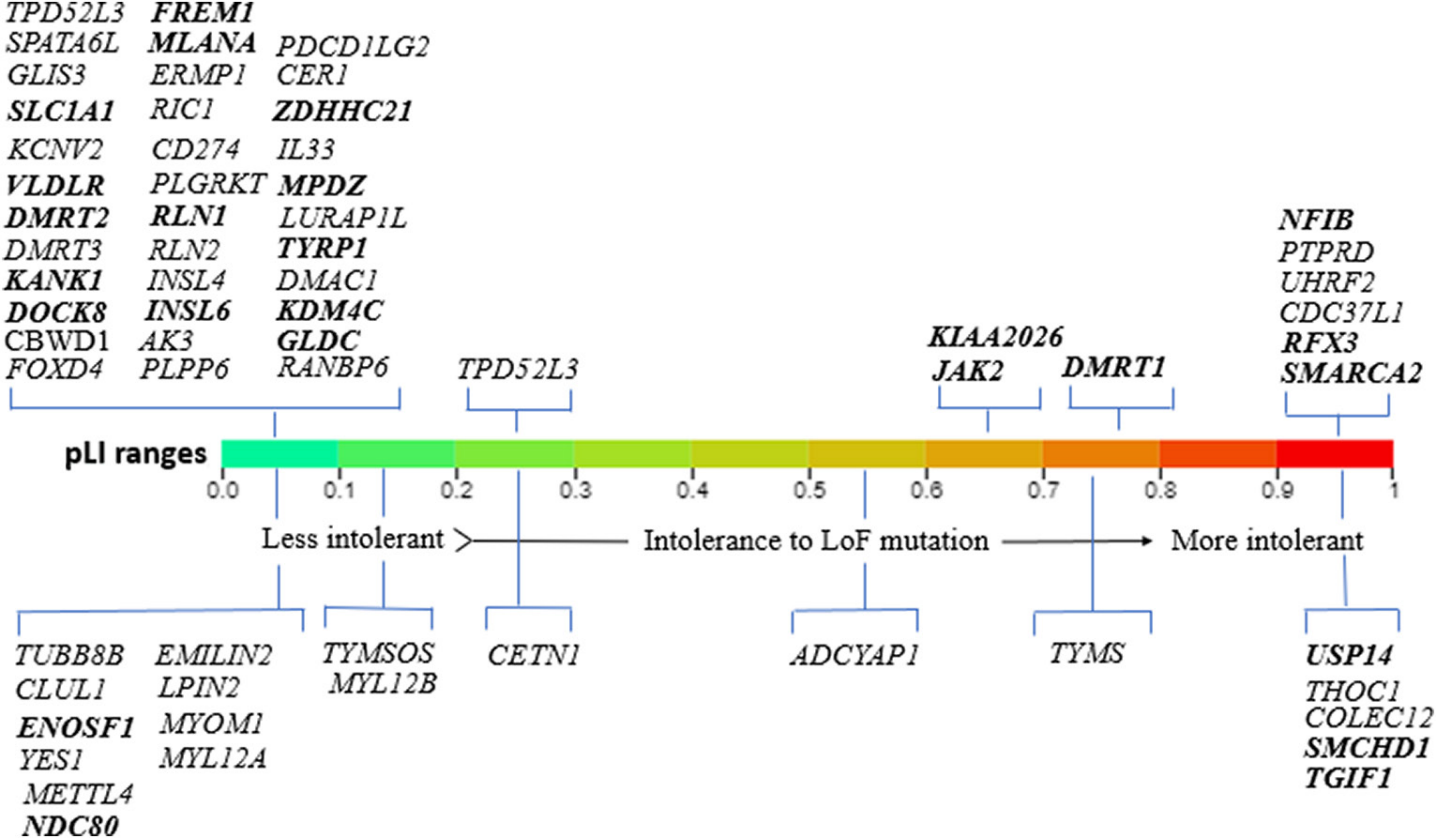
Affected genes with pLI score for 9p22.3‐p24.3 deletion (above panel) and 18p11.31‐p11.32 (below panel). Highlighted genes represent genes that phenotyping matching have been done for them.

Based on including criteria for overlapping CNVs, the 18p11.31‐p11.32 region had 19 overlapping CNVs in the DECIPHER with the size ranging from 23.42 kb to 1.82 Mb (Table S2) These CNVs affect six intolerant genes (high pLI genes) *TGIF1*, *COLEC12*, *SMCHD1*, *THOC1*, *TYMS*, and *USP14* (Tables 1 and S3). Figure 2 represents an illustration of the affected genes within 9p22.3p24.3 and 18p11.31‐p11.32 for which pLI score have been reported.

## 4 DISCUSSION

Cytogenetic and molecular analyses of the present case confirmed a 46,XY,der(9),t(9;18)(p22;p11.31)mat and revealed a 15.056 Mb deletion of the 9p22.3‐p24.3 combined with a 3.309 Mb duplication of the 18p11.31‐p11.32 due to the unbalanced segregation of a maternal balanced reciprocal translocation. The proband's brother also presented to the clinic for an evaluation of developmental delay and cytogenetic analyses showed the same aberration for karyotypic male. Like his sister, he also had clinical manifestations typical of monosomy 9p syndrome. Here we systematically present the PhenogramViz matching outputs along with characterization of genes that overlap the detected CNVs using DECIPHER data.

### 4.1 Neurodevelopmental and neuroimaging phenotypes

**FIGURE 3:**
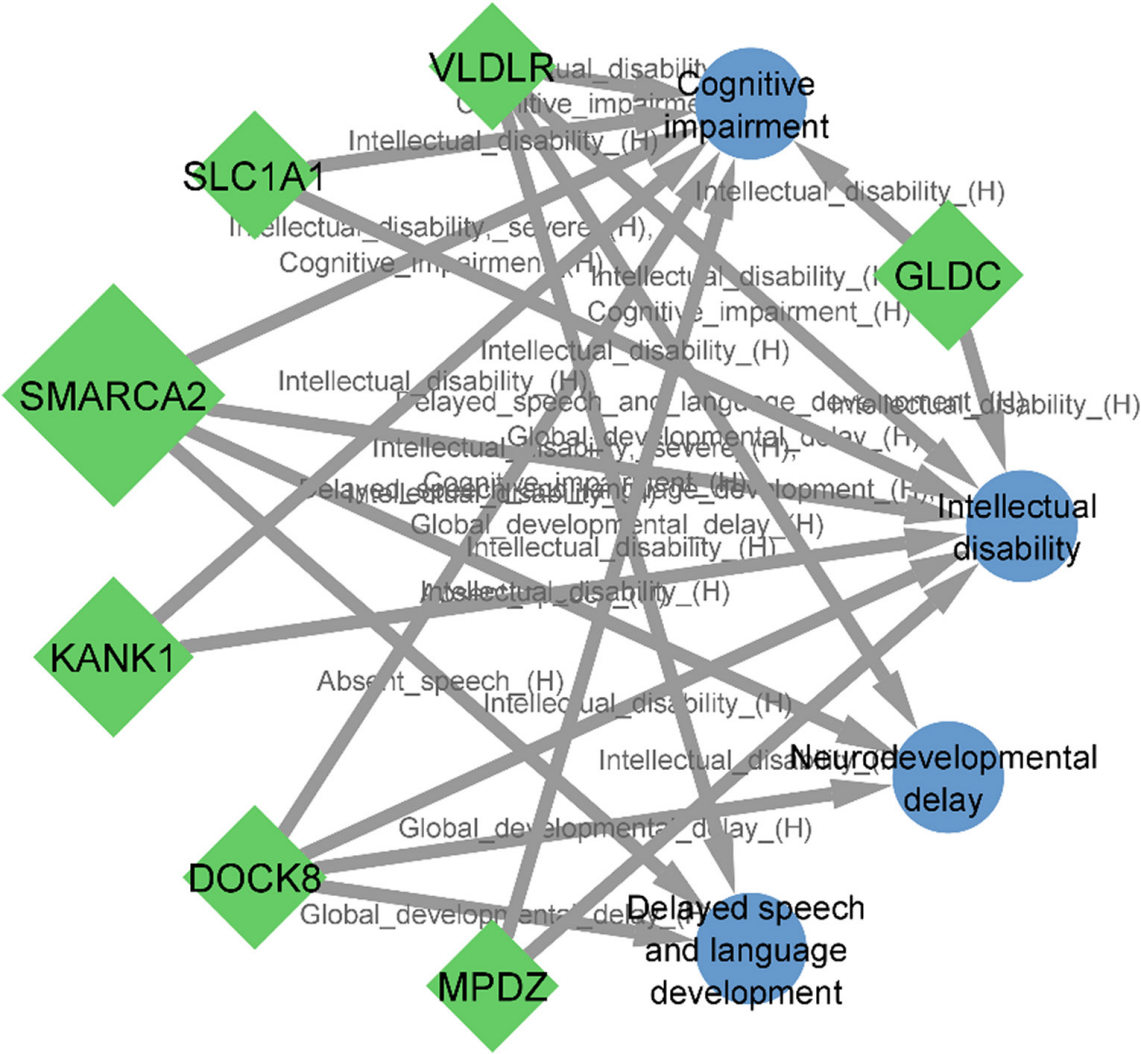
Phenogram for neurodevelopmental features showing matching of seven genes. The size of a gene node is proportional to its haploinsufficiency score.

For matching the neurodevelopmental features of our patients to affected genes within detected CNVs, we used the HPO terms, intellectual disability, neurodevelopmental delay, cognitive impairment, delayed speech and language development. Seven genes on the 9p22.3‐p24.3, *SMARCA2*, *DOCK8*, *KANK1*, *VLDLR*, *MPDZ*, *SLC1A1* and *GLDC* were matched to neurodevelopmental phenotypes, but no matching was seen for genes located in 18p11.31‐p11.32 (Figure 3).

**FIGURE 4:**
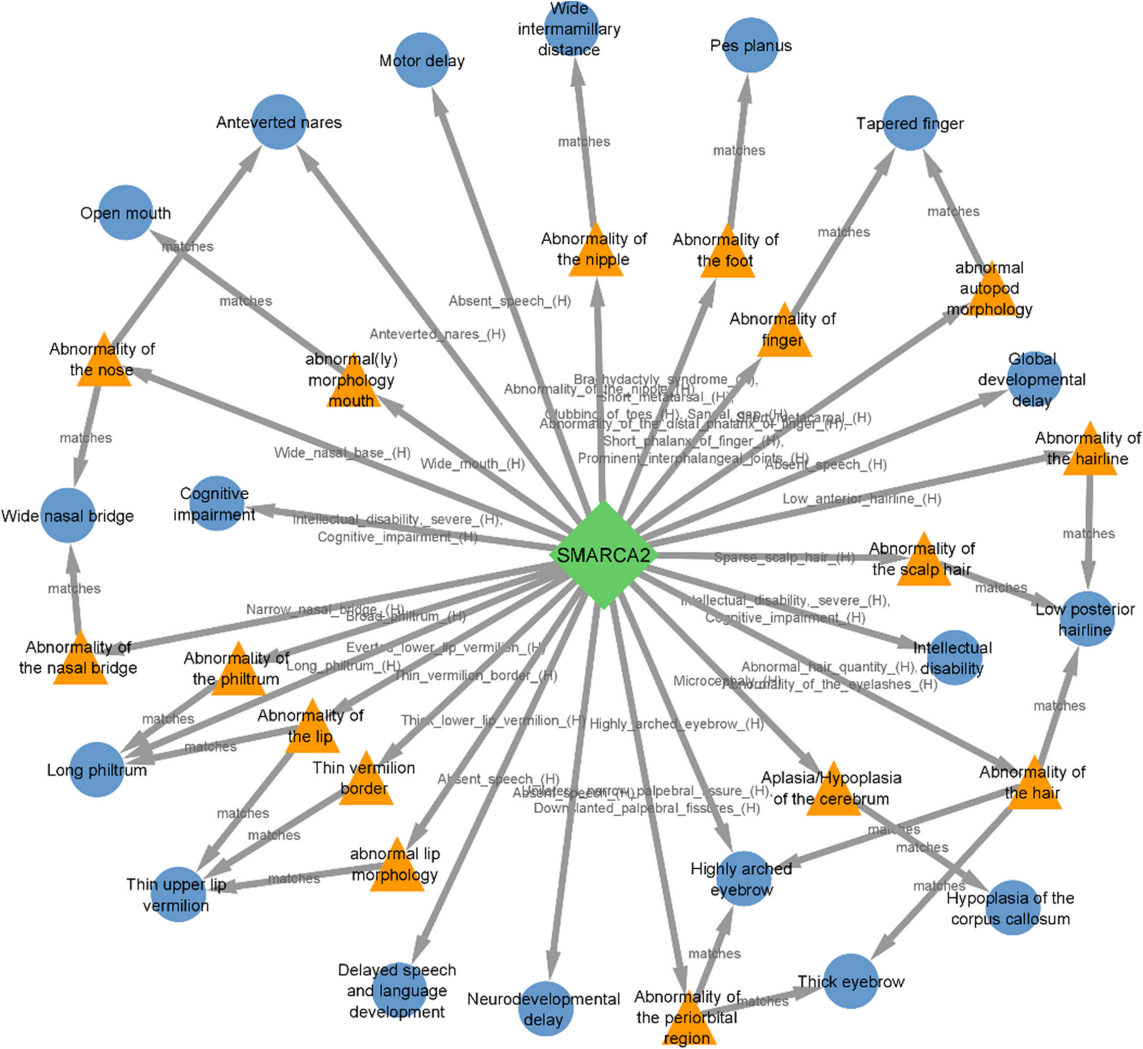
Phenogram for phenotyping matching of *SMARCA2* gene showing matching of 18 phenotypes. The common ancestors between a gene's phenotype annotation and the patient's phenotypes are displayed as orange triangles.

*SMARCA2* is an intolerant gene to loss‐of‐function variants (pLI = 1, Figure 4) and also to haploinsufficiency mechanisms (score = 1.60), was directly or indirectly matched to 18 phenotypes of our cases indicating the critical role of this gene in clinical manifestation of 9p deletion syndrome (Figure 4). Mutations and deletions in this gene have been reported to be responsible for the Nicolaides–Baraitser syndrome (OMIM 601358) that is characterized by ID, seizures, microcephaly, sparse hair, short stature, typical face, brachydactyly, and behavioural problems.^13^

Heterozygous mutations and deletions in the *DOCK8* gene have been reported in individuals affected with autosomal dominant ID^14^ and deletion of the *KANK1* gene has been associated with congenital cerebral palsy, hypotonia and ID.^15^ *VLDLR* is another critical gene in the 9p24.3 region, mutations in which can cause cerebellar ataxia, ID and disequilibrium syndrome 1.^16^ Above mentioned information together with gene–phenotype matching using PhenogramViz can support that haploinsufficiency of the *DOCK8*, *KANK1* and *VLDLR* may be responsible for neurological manifestation in individuals with 9p deletion syndrome (Figure S6).

**FIGURE 5:**
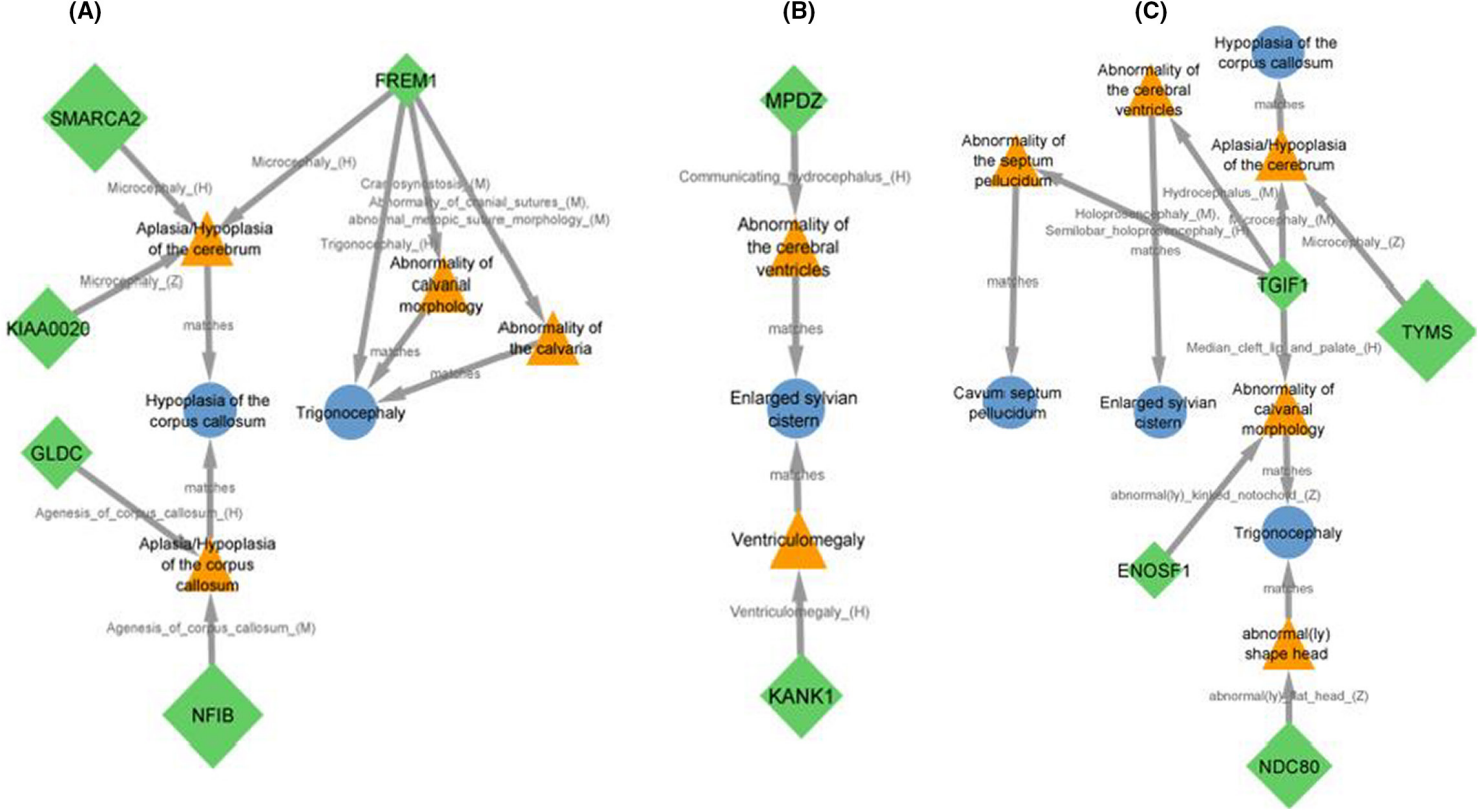
Phenogram for Brain MRI findings, hypoplasia of corpus callosum, enlarged sylvian cistern, and trigonocephaly showing matching of seven genes. For cavum septum pellucidum and delayed myelination, no gene matching was found. (A and B) show genes located at 9p22.3‐p24.3 and (C) show genes located at 18p11.31‐p11.32.

For brain MRI findings, seven genes on 9p, and four genes on 18p were matched to hypoplasia of corpus callosum (*SMARCA2*, *KIAA0020*, *NFIB*, *GLDC* and *FREM1* located at 9p and *TGIF1*, *TYMS*, *ENOSF1* located at 18p, Figure 5A,C), cavum septum pellucidum (*TGIF1*, Figure 5C), enlarged sylvian cistern (*KANK1* and *MPZ* located at 9p and *TGIF1*, Figure 5B,C), delayed myelination (no matching was found) and trigonocephaly (*FREM1*, *TGIF1*, *ENOSF1* and *NDC80*, Figure 5A,C).

In support of the role of the *KANK1* gene, in a study by Lerer et al.,^17^ neuroimaging for nine children belonging to a four‐generation family with deletion of the *KANK1* gene showed brain atrophy and ventriculomegaly. Hypothetically, ventriculomegaly may be act as a common ancestor phenotype between *KANK1* and enlarged sylvian cistern. Accumulation of CSF in the ventricles due to mutations in the *MPDZ* gene may be also cause ventriculomegaly.^18^ The other potential disease‐associated gene for the patient 2's observed neuroimaging phenotypes is *TGIF1* located at 18p11.3. As a dosage‐sensitive gene, *TGIF1* was matched to four neuroimaging phenotypes including hypoplasia of corpus callosum, cavum septum pellucidum, enlarged sylvian cistern, and trigonocephaly.

Trigonocephaly as a notable craniofacial feature in our patients (Figures 1 and S1) was matched also to *FREM1* gene, which resides in the 300 kb interval on 9p24.3 region. This segment for the first time was defined by Swinkels et al.^19^ as a candidate for metopic craniosynostosis, the early fusion of the frontal bones that results in trigonocephaly as a prominent feature of 9p deletion syndrome.

### 4.2 Facial phenotypes

Six different facial phenotypes including hypertelorism, wide nasal bridge, anteverted nostrils, long philtrum, thin upper vermilion and the open mouth were matched to the five genes on 9p, *SMARCA2*, *JAK2*, *NFIB*, *KDM4C* and *FREM1* (Figure S7) and two genes on 18p, *SMCHD1* and *TGIF1* (Figure S8). Notable similarities in facial appearance with epicanthal folds, highly arched eyebrows, sparse eyelashes, broad nasal bridge, downturned nasal tip in individuals carrying *SMARCA2* variants have been reported by Cappuccio et al.,^20^ that indicates the clinical importance of small critical region encompassing *SMARCA2* on 9p24.3 region (Figure 4).

### 4.3 Genital phenotypes

Genital abnormalities are estimated to be present in up to 70% of patients with partial 9p monosomy.^21^ The DMRT genes (*DMRT1*, *DMRT2* and *DMRT3*) located at 9p24.3 as a critical interval for 46,XY complete gonadal dysgenesis and haploinsufficiency of them may cause gonadal dysgenesis as well as abnormal external genitalia, especially in karyotypic males.^22^ Cryptorchidism and large penis were present in patient 2, but sex reversal was not observed in her. Abnormal external genitalia were not present in patient 1. Genitourinary system findings in our cases may reflect variable penetrance and expressivity of *DMRT1*. However, the matching of *DMRT1*, *DMRT2* to abnormality of the male external genitalia and large penis by PhenogramViz (Figure S9) is in line with phenotypic and functional consequences of haploinsufficiency of *DMRT* genes. In addition to *DMRT* genes, *RLN1* and *INSL6* are two other genes that have been matched to abnormality of male external genitalia (Figure S9). Co‐occurring of these two genes with the cryptorchidism in abstracts of biomedical publications from the DISEASES Text‐mining Gene‐Disease Association Evidence Scores dataset have been reported by Harmonizome with relatively high scores.^23^

In summary, the major clinical features of chromosome distal 9p deletion syndrome (OMIM 158170) were seen in patients harbouring deleted CNVs smaller than 1 Mb in size in the 9p24.3 region. This segment of 9p as a hot spot of copy number losses may be responsible for major clinical manifestation of this syndrome.^16^, ^24^ In support of this hypothesis are clinical implications of critical genes within this region such as 46,XY gonadal dysgenesis and 46,XY ovotesticular disorder of sexual development (haploinsufficiency of *DMRT1*),^25^ autosomal dominant ID 2 (heterozygous disruption of *DOCK8* either by a translocation breakpoint or a deletion),^14^ familial cerebral palsy (deletion of *KANK1*)^17^ and cerebellar hypoplasia and ID with or without quadrupedal locomotion 1 (Mutations of *VLDLR*).^26^ Based on DECIPHER search, eight genes with a pLI score were in the 9p24.3 region including *CBWD1*, *FOXD4*, *DOCK8*, *KANK1*, *DMRT1*, *DMRT*2, *DMRT*3 and *SMARCA2* that three of which, *DOCK8*, *KANK1 and SMARCA2* are OMIM morbid genes. like *SMARCA2* as a multiple phenotype matched gene, *TGIF1* matched to 10 phenotypes through ancestor phenotypes (Figure S10). Among these phenotypes, ventricular septal defect is notable in that it is matched directly to *RFX3* located at 9p24.2.

Interestingly, 34 of 80 DECIPHER cases have CNVs involving brain overexpressed gene, *PTPRD*. 18 of them harbour only this gene that is intolerant to loss of function variants (pLI = 1) and also to haploinsufficiency mechanisms (score = 0.14). Hypothetically, having an additive effect in causing patient's clinical manifestations especially autistic behaviour could be attributed to *PTPRD*. Recently implicated as a candidate gene for restless legs syndrome^27^ and associated with neurodevelopmental disorders,^28^ *PTPRD* may present with ADHD and autistic behaviours; however, it is not associated with a disorder in the OMIM database, indicating that its implication in human disease still required further clarification.^28^ Report of patients with ID and trigonocephaly harbouring homozygous deletions of *PTPRD* by Choucair et al.^29^ is another supporting finding for the role of *PTPRD* in neurodevelopmental disorders. In the output of PhenogramViz, this gene was not matched to any clinical manifestation of our patients.

In conclusion, this study presents an overview of cytogenetic aberrations, developmental features, and clinical characteristics in two patients presenting with 9p22.3‐p24.3 and duplication of 18p11.31‐p11.32. Furthermore, in order to identify the gene or genes located in detected CNV regions that could potentially act as genetic modifiers of developmental features and clinical characteristics of our patients, the PhenogramViz plugin of CytoScape was used. Finally, for finding specific gene–phenotype associations and narrowing the critical region for the deletion 9p syndrome, overlapping CNVs were sought using DECIPHER database. It should be noted that although aCGH has greatly advanced the identification of CNVs in the human genome, mechanisms of origin of rearrangements are often difficult to resolve, and predicting their effects on gene expression and phenotype remains a challenge. Beside gene–phenotype characterizations via bioinformatic analysis, an important message of this study is that aCGH is a useful approach for diagnosis of the patients with neurodevelopmental delay/intellectual disability and dysmorphic features born to non‐consanguineous parents.

## AUTHOR CONTRIBUTIONS

**Naser Ajami:** Conceptualization (equal); data curation (equal); formal analysis (equal); writing – original draft (equal). **Mohammad Amin Kerachian:** Investigation (equal); resources (equal); writing – review and editing (supporting). **Mehran Beiraghi Toosi:** Resources (equal); validation (equal); writing – review and editing (supporting). **Farah Ashrafzadeh:** Resources (equal); validation (equal); writing – review and editing (supporting). **Susan Hosseini:** Methodology (equal); resources (supporting); writing – review and editing (supporting). **Peter N. Robinson:** Conceptualization (equal); funding acquisition (equal); software (equal); supervision (equal); writing – review and editing (equal). **Mohammad Reza Abbaszadegan:** Funding acquisition (equal); supervision (equal); validation (equal); visualization (equal); writing – review and editing (equal).

## FUNDING INFORMATION

This study was supported by NIH NHGRI U24HG011449 and Medical Genetics Research Center (MGRC), Mashhad University of Medical Sciences, Mashhad, Iran.

## CONFLICT OF INTEREST

The authors report no conflict of interest relevant to the manuscript.

## Supporting information


Figures S1–S10.

Table S1.

Table S2.

Table S3.

## Data Availability

Data available on request from the authors.
