# Supplementary material for: Inherited deletion of 9p22.3‐p24.3 and duplication of 18p11.31‐p11.32 associated with neurodevelopmental delay: Phenotypic matching of involved genes
Source: J Cell Mol Med. 2023 Jan 24;27(4):496–505. doi: 10.1111/jcmm.17662 (PMC9930415; doi:10.1111/jcmm.17662)
Supplement: Supplementary file 1 — Figures S1–S10. [file JCMM-27-496-s002.docx]

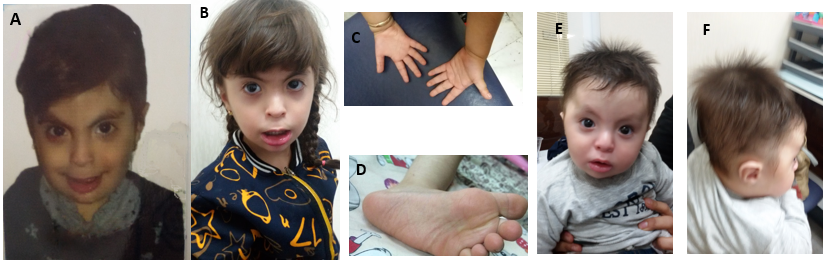


Supp. figure 1. A and B. Frontal view of the Patient 1 at the age of 3.5-year-old showing a prominent vertical ridge in the center of the forehead reflecting trigonocephalic head of the patient, highly arched eyebrow, wide nasal bridge, long philtrum, thin upper lip vermilion, open mouth, downturned corners of the mouth, C. tapering fingers, D. rocker-bottom feet, E and F, Frontal and lateral views of the patient 2. trigonocephalic flat head is considerable in patient 2 (F)


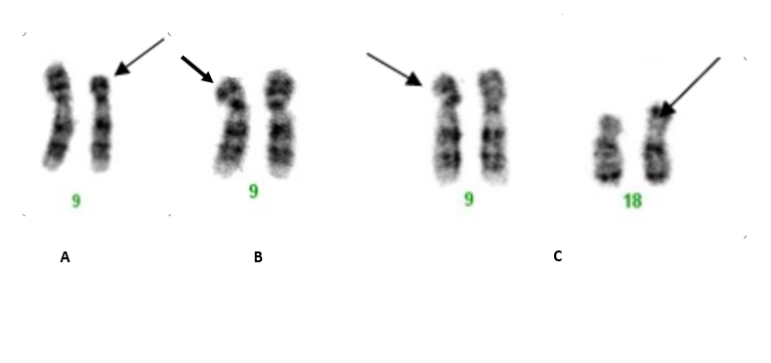


Supp. Figure 2. G-banded partial karyotype of the patient 1 (A), patient 2 (B) and their mother (C) showing a terminal deletion of chromosome 9 and t(9;18)(p22;p11.31) respectively.


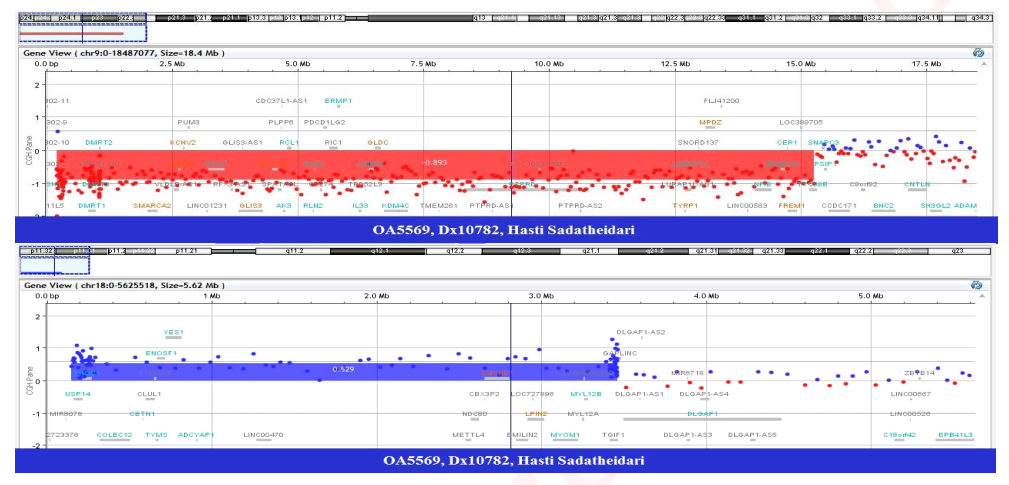


Supp. Figure 3. aCGH analysis for patient 1 shows 15.056Mb deletion of the chromosome 9 (9p24.3p22.3 region) and 3.309 Mb duplication of the chromosome 18 (18p11.32p11.31).


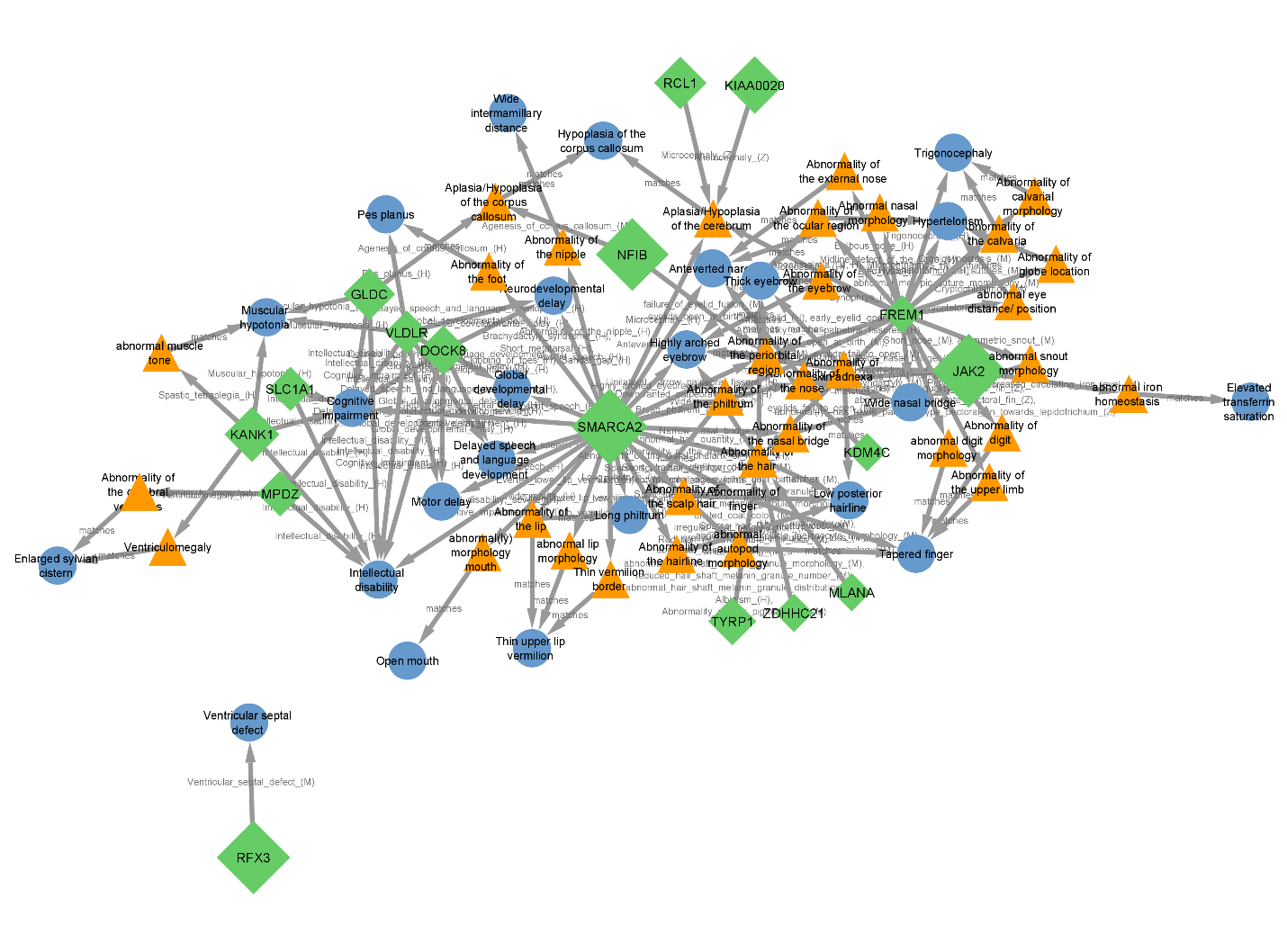


Supp. figure 4. Phenotypic matching for 9p24.3p22.3.


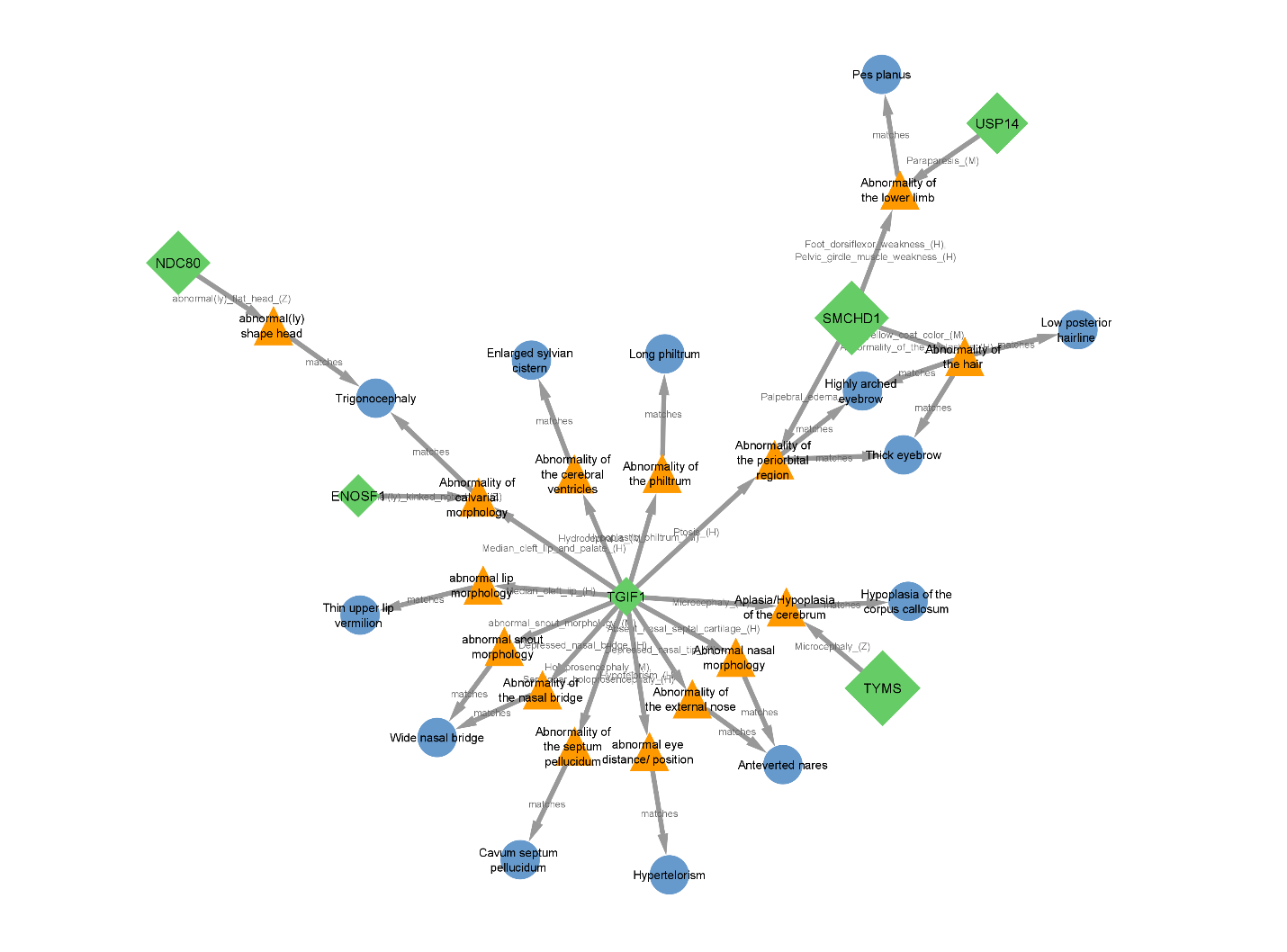


Supp. figure 5. Phenotypic matching for 18p11.32p11.31


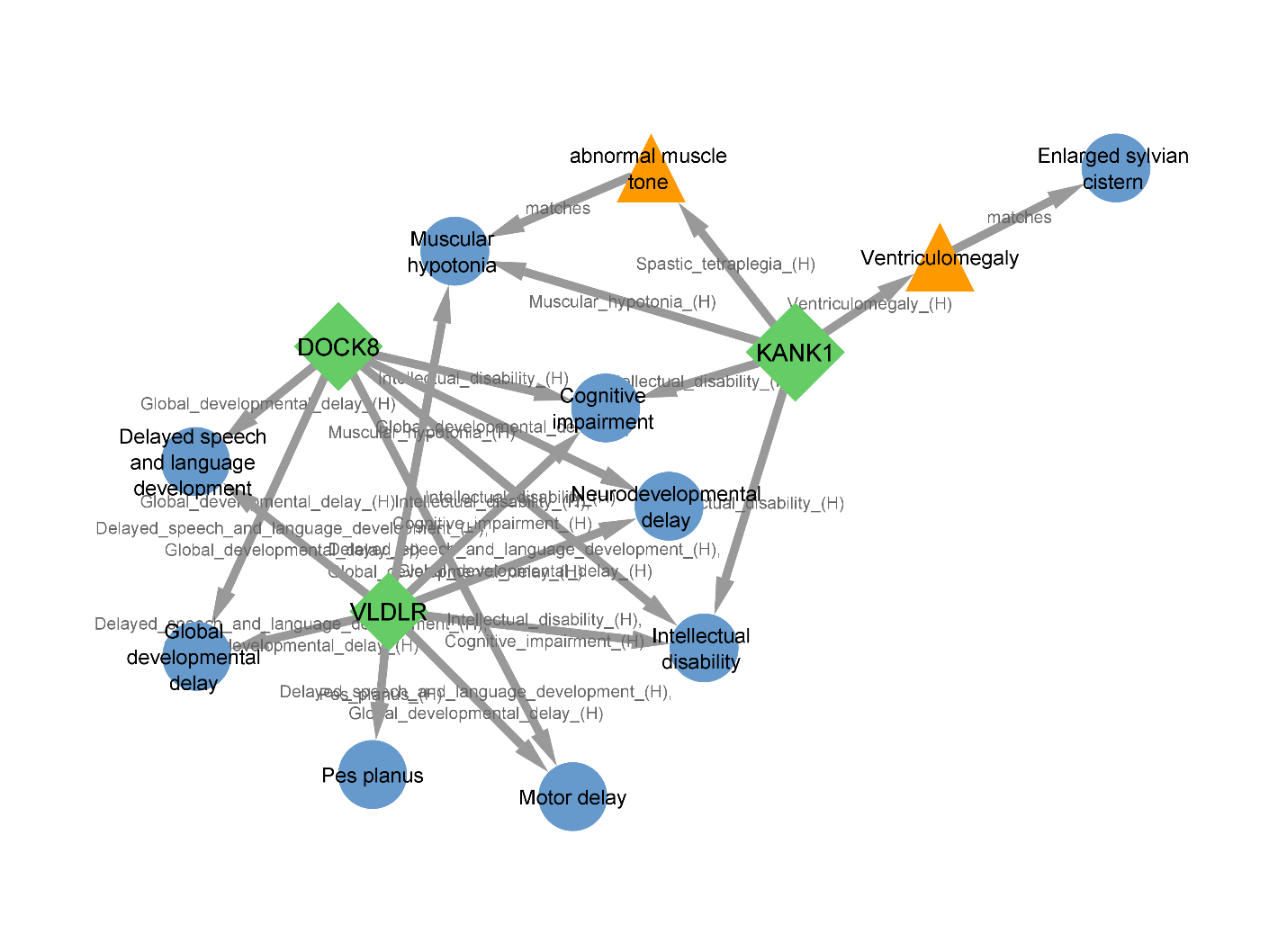


Supp. Figure 6. Phenogram for *VLDR*, *DOCK8*, and *KANK1*


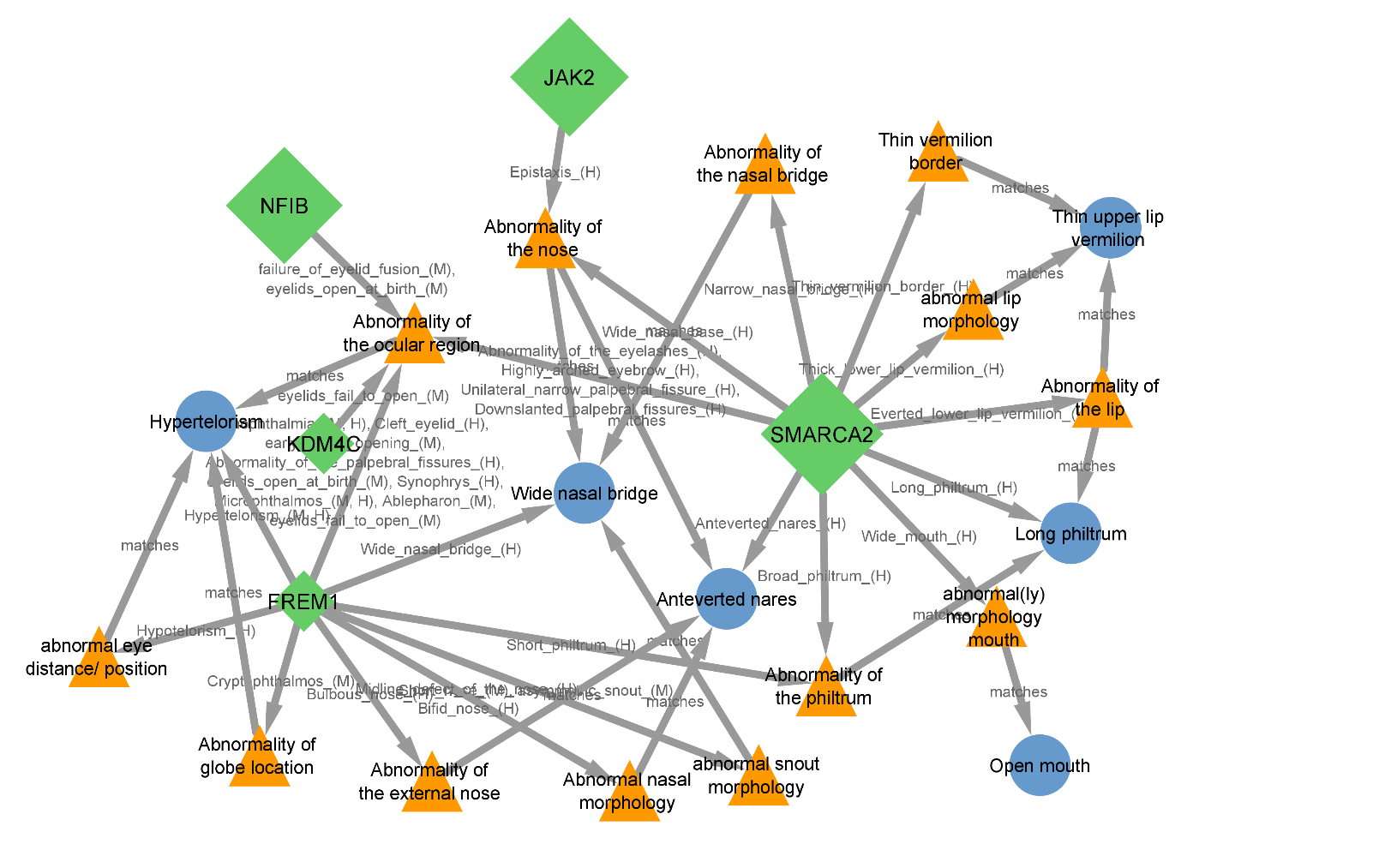


Supp. Figure 7. Phenogram for facial phenotypes that matched to genes located at 9p24.3p22.3.


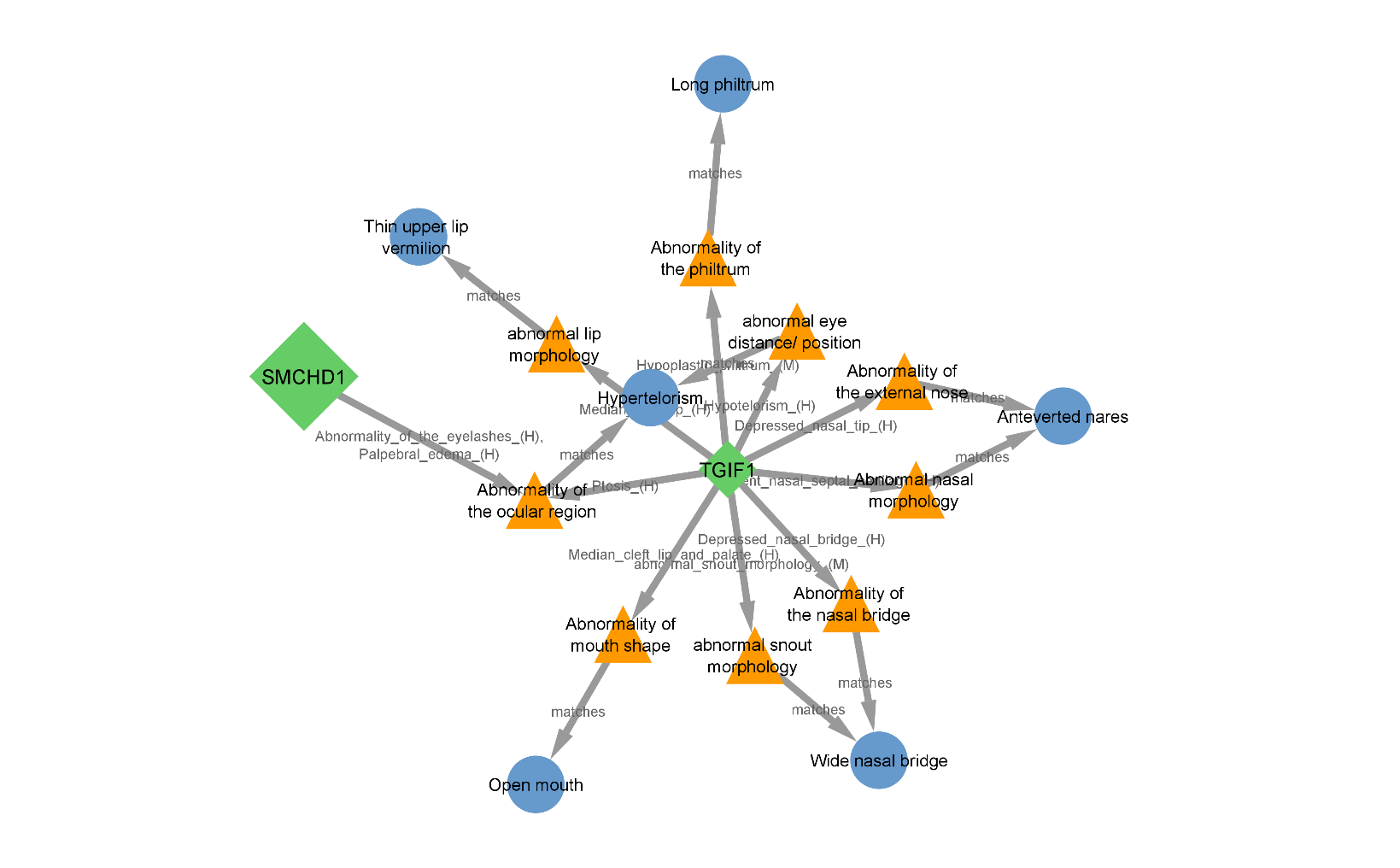


Supp. Figure 8. Phenogram for facial phenotypes that matched to genes located at 18p11.32p11.31.


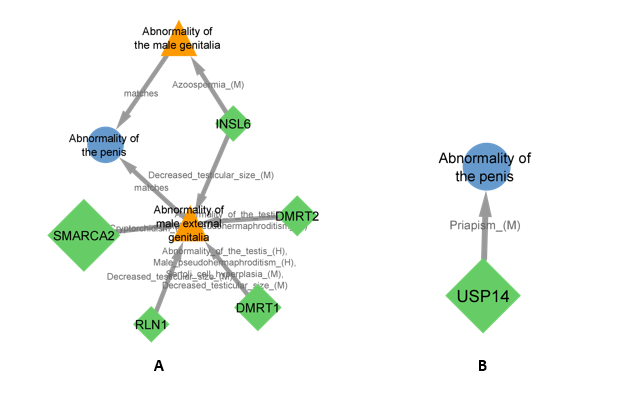


Supp. Figure 9. Phenogram for abnormality of the penis showing matching of four genes on 9p and three genes on 18p.


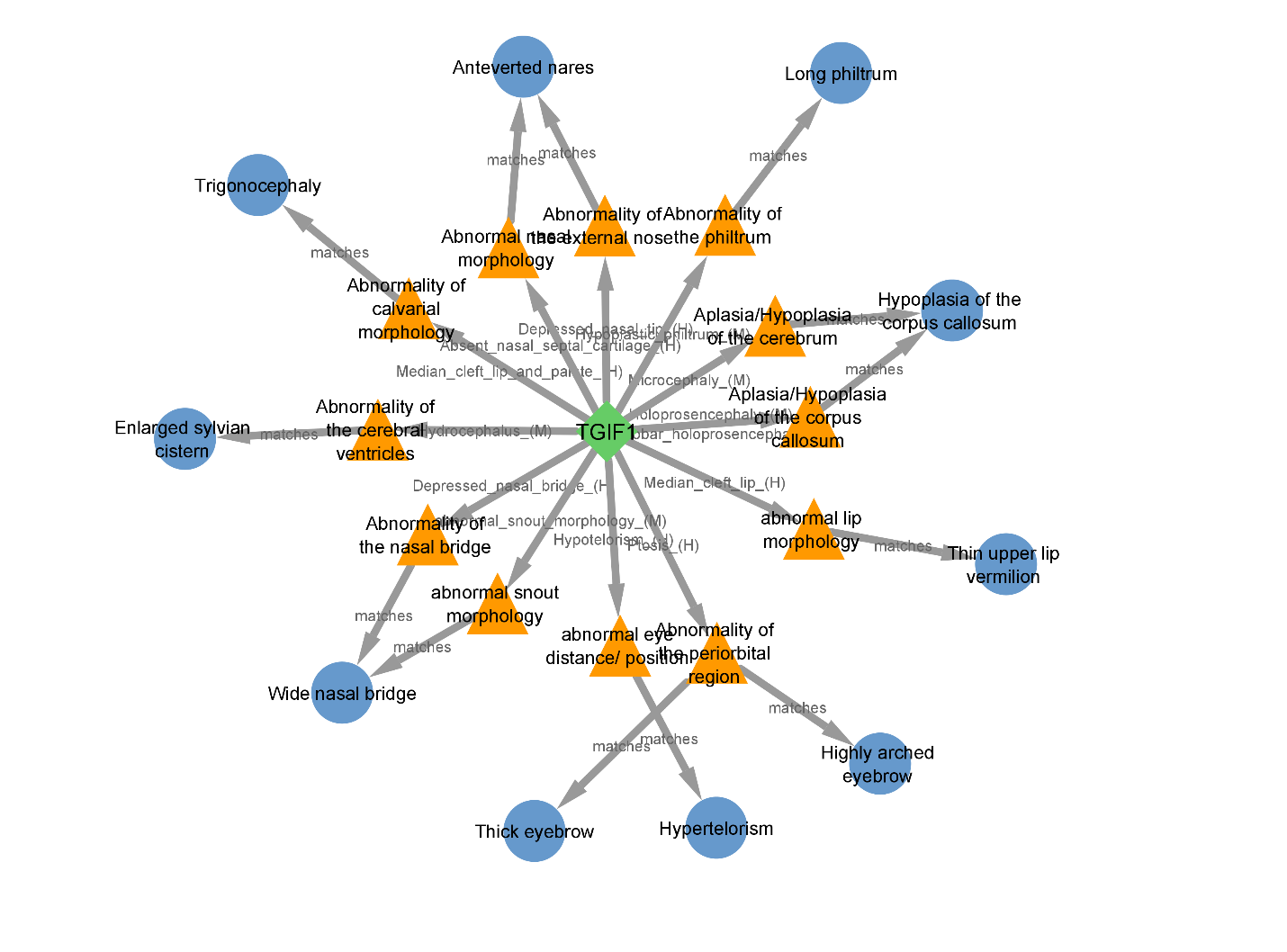


Supp. Figure 10. Phenogram for TGIF1
