# Supplementary material for: Inherited deletion of 9p22.3‐p24.3 and duplication of 18p11.31‐p11.32 associated with neurodevelopmental delay: Phenotypic matching of involved genes
Source: J Cell Mol Med. 2023 Jan 24;27(4):496–505. doi: 10.1111/jcmm.17662 (PMC9930415; doi:10.1111/jcmm.17662)
Supplement: Supplementary file 2 — Table S1. [file JCMM-27-496-s003.pdf]

**DECIPHER Patient**	**Sex**	**Location**	**Size**	**Inheritance / Genotype**
380672	46XX	14116843-14128849	12.01 kb	De novo Heterozygous
257660	46XX	928142-952334	24.19 kb	Unknown Heterozygous
304981	46XY	14865548-14915743	50.20 kb	Unknown Heterozygous
289419	46XX	204090-254781	50.69 kb	Unknown Heterozygous
287929	Unknown	6610913-6667926	57.01 kb	Maternally inherited Heterozygous
294652	46XY	9905054-9972076	67.02 kb	Unknown Heterozygous
343435	46XY	500525-569970	69.45 kb	Unknown Heterozygous
331458	46XY	182650-254781	72.13 kb	Unknown Heterozygous
278454	46XY	617082-698729	81.65 kb	Unknown Heterozygous
355295	46XY	9972017-10060074	88.06 kb	Unknown Heterozygous
325756	46XY	9972017-10060074	88.06 kb	Unknown Heterozygous
250594	46XY	2688899-2788254	99.36 kb	Unknown

				Heterozygous
299480	46XX	9872103-9972047	99.94 kb	Biparental Homozygous
340337	46XY	9043312-9150024	106.71 kb	Unknown Heterozygous
278647	46XY	6531227-6655056	123.83 kb	Paternally inherited Heterozygous
318367	46XY	204090-329711	125.62 kb	Unknown Heterozygous
307787	46XX	6230592-6359510	128.92 kb	Unknown Heterozygous
359510	46XX	4002486-4135134	132.65 kb	Unknown Heterozygous
338679	46XX	9577040-9713032	135.99 kb	Unknown Heterozygous
268839	46XX	7042590-7180796	138.21 kb	Unknown Heterozygous
325823	46XY	9363262-9514361	151.10 kb	Unknown Heterozygous
338694	46XX	9972017-10126280	154.26 kb	Unknown Heterozygous
275647	46XY	902048-1057701	155.65 kb	Unknown Heterozygous

262846	46XY	5670381-5830137	159.76 kb	De novo<br><br>Heterozygous
275450	46XY	8364878-8528849	163.97 kb	Unknown<br>Heterozygous
331297	46XY	14853637-15041023	187.39 kb	Maternally inherited<br>Heterozygous
369930	46XY	9872073-10060074	188.00 kb	Unknown<br>Heterozygous
291053	46XY	8842562-9043311	200.75 kb	Unknown<br>Heterozygous
381984	46XY	579790-789845	210.06 kb	Paternally inherited<br><br>Heterozygous
407877	Unknown	12693407-12907827	214.42 kb	Unknown<br><br>Heterozygous

404135	46XY	9320906-9537667	216.76 kb	Unknown<br><br>Heterozygous
411682	46XY	14483292-14700359	217.07 kb	Unknown Heterozygous
427307	46XY	730054-1002280	272.23 kb	De novo Heterozygous
255468	46XX	6055497-6330726	275.23 kb	Inherited from normal parent<br><br>Heterozygous
277952	46XY	6055497-6330726	275.23 kb	Paternally inherited Heterozygous
306888	46XY	9957129-10236864	279.74 kb	Maternally inherited Heterozygous
410313	46XY	9195377-9486652	291.28 kb	Unknown<br><br>Heterozygous
278247	46XY	203993-500725	296.73 kb	Inherited from normal parent Heterozygous

331275	46XX	11356040-11659349	303.31 kb	Maternally inherited Heterozygous
349604	46XY	10164896-10473327	308.43 kb	Unknown Heterozygous
366485	46XY	11952522-12275177	322.66 kb	Unknown Heterozygous
331491	46XY	11318956-11659349	340.39 kb	Unknown Heterozygous
318804	46XY	7048808-7406813	358.01 kb	Unknown Heterozygous
294452	46XY	9872073-10286986	414.91 kb	Paternally inherited Heterozygous
270837	46XY	9282591-9735948	453.36 kb	Inherited from normal parent Heterozygous
265424	46XY	6848731-7329484	480.75 kb	Inherited from normal parent Heterozygous

401706	46XY	4884849-5398000	513.15 kb	De novo<br>Heterozygous
407059	46XX	204193-730088	525.90 kb	Unknown<br>Heterozygous
323730	46XY	9514302-10164955	650.65 kb	Unknown<br>Heterozygous
331085	46XX	6055502-6706559	651.06 kb	Paternally inherited<br>Heterozygous
293478	46XX	639010-1411809	772.80 kb	De novo<br>Heterozygous

401728	46XX	13155536-14115572	960.04 kb	De novo<br>Heterozygous
262062	46XY	183890-1783890	1.60 Mb	Unknown<br>Heterozygous
322014	46XY	11775361-14110243	2.33 Mb	Unknown<br>Heterozygous
261817	46XY	11457340-14253280	2.80 Mb	Unknown<br>Heterozygous
277068	46XY	12201782-15517595	3.32 Mb	De novo<br>Heterozygous
367212	46XX	204193-3662436	3.46 Mb	De novo<br>Heterozygous

278243	46XX	2547415-6198739	3.65 Mb	De novo Heterozygous
351576	46XX	204090-3965567	3.76 Mb	Unknown Heterozygous
401659	46XY	203862-4094552	3.89 Mb	De novo Heterozygous
288608	Unknown	6494182-10610832	4.12 Mb	Maternally inherited Heterozygous
266308	46XX	271230-4768803	4.50 Mb	De novo Heterozygous

396066	46XX	9010000-14110001	5.10 Mb	Unknown<br><br>Heterozygous
396067	46XX	9010000-14110001	5.10 Mb	Unknown<br><br>Heterozygous
396100	46XY	9010000-14110001	5.10 Mb	Imbalance arising from a balanced parental rearrangement<br><br>Heterozygous
280384	46XX	271257-5558593	5.29 Mb	Unknown<br>Heterozygous

433840	46XY	211086-5881488	5.67 Mb	Unknown Heterozygous
281904	46XY	611628-6526211	5.91 Mb	De novo Heterozygous
4434	46XY	8680463-14910945	6.23 Mb	De novo Heterozygous
257497	46XX	214367-7202935	6.99 Mb	De novo Heterozygous
280534	46XY	204193-8361148	8.16 Mb	Unknown Heterozygous

300606	Unknown	611628-8842561	8.23 Mb	Unknown Heterozygous
276146	46XY	3063252-11573652	8.51 Mb	De novo<br>Heterozygous
407886	Unknown	271257-8935571	8.66 Mb	Unknown Heterozygous

262434	46XX	204221-9735778	9.53 Mb	De novo<br>Heterozygous
301243	46XX	208454-9737404	9.53 Mb	De novo<br>Heterozygous
250657	46XY	2418075-14025666	11.61 Mb	De novo<br>Heterozygous

265978	46XY	204193-11868583	11.66 Mb	Unknown Heterozygous
257033	46XX	204220-15549762	15.35 Mb	De novo Heterozygous
428963	46XY	204193-18654814	18.45 Mb	De novo Heterozygous

**Pathogenicity / Contribution**	**Phenotype(s)**	**Morbid genes**	**High pLI genes**
	Mild global developmental delay	*NFIB*	*NFIB*
	Intellectual disability		*DMRT1*
Uncertain<br>Uncertain	Macrocephaly	*FREM1*	
Uncertain	Global developmental delay	*DOCK8*	
Uncertain	Nonketotic hypoglycemia	*GLDC*	
Likely pathogenic	Autistic behavior		*PTPRD*
Uncertain	Autistic behavior	*KANK1*	
Likely pathogenic	Behavioral abnormality, Intellectual disability, mild, Mood swings	*DOCK8*	
Uncertain	Behavioral abnormality, Febrile seizure (within the age range of 3 months to 6 years), Global developmental delay	*KANK1*	
Uncertain	Autism, Global developmental delay		*PTPRD*
Uncertain<br>Uncertain	Abnormal emotion/affect behavior		*PTPRD*
	Deeply set eye, Depression, Intellectual disability	*KCNV2*	

	Intellectual disability		
Uncertain	Growth delay		*PTPRD*
	Autism		*PTPRD*
	Motor delay, Specific learning disability	*GLDC*	
	Abnormal emotion/affect behavior	*DOCK8*	
Uncertain	Cognitive impairment		
Uncertain	Arachnodactyly, Hypertelorism, Pointed chin, Preauricular skin tag, Proptosis, Retrognathia	*GLIS3*	
Uncertain	Intellectual disability		*PTPRD*
	Plagiocephaly, Psychosis		
Uncertain<br>Uncertain	Abnormal emotion/affect behavior, Delayed speech and language development		*PTPRD*
Uncertain	Delayed speech and language development		*PTPRD*
	Ovarian gonadoblastoma		

	Cryptorchidism, Hydronephrosis, Hypospadias, Microcephaly, Proportionate short stature, Small for gestational age		
	Abnormality of higher mental function		*PTPRD*
Likely pathogenic	Abnormality of the head, Abnormality of tibia morphology	*FREM1*	
Uncertain	Cognitive impairment, Schizophrenia		*PTPRD*
Uncertain	Autistic behavior, Global developmental delay		*PTPRD*
Uncertain<br>Uncertain	Cortical dysplasia, Hypotonia, Motor delay, Optic nerve hypoplasia, Panhypopituitarism, Polymicrogyria, Tall stature	*KANK1*	
Uncertain<br>Partial	Albinism, Congenital bullous ichthyosiform erythroderma, Iris transillumination defect, Myopia, Nevus, Ocular albinism, Photophobia, Unilateral renal hypoplasia, Vesicoureteral reflux	*TYRP1*	

Uncertain<br><br>Uncertain	Delayed speech and language development, Dyscalculia, Dysgraphia, Poor fine motor coordination, Specific learning disability		*PTPRD*
Uncertain<br>Uncertain	Autism, Intellectual disability	*FREM1*	
	Macrocephaly, Ventricular septal defect		*DMRT1*
	Behavioral abnormality, Everted lower lip vermilion, Frontal bossing, High anterior hairline, Hypoplasia of the corpus callosum, Intellectual disability, Long face, Posteriorly rotated ears		
	Intellectual disability, Microcephaly		
Uncertain<br>Uncertain	Cognitive impairment		*PTPRD*
Uncertain<br>Uncertain	Attention deficit hyperactivity disorder, Behavioral abnormality, Obesity		*PTPRD*
	Intellectual disability, mild, Strabismus	*DOCK8*<br>*KANK1*	

Likely pathogenic	Failure to thrive, Low-set ears		
Uncertain	Behavioral abnormality		*PTPRD*
Uncertain	Cognitive impairment		
Likely pathogenic	Intellectual disability, mild, Specific learning disability		
Uncertain<br>Uncertain	Global developmental delay		
Uncertain	Autistic behavior, Dysphasia, Obesity		*PTPRD*
	Autism, Seizure		*PTPRD*
	Abnormal hair whorl, Intellectual disability, Uplifted earlobe		

Likely pathogenic	Abnormality of the lower limb, Caesarian section, Feeding difficulties in infancy, Hearing abnormality, Long eyelashes, Long phalanx of finger, Long toe, Neonatal asphyxia, Premature birth, Proportionate short stature, Short stature, Small for gestational age, Synophrys	*JAK2*	
Likely pathogenic<br>Partial		*DOCK8*<br>*KANK1*	
Uncertain<br><br>Uncertain	Abnormal facial shape, Exocrine pancreatic insufficiency, Recurrent respiratory infections		*PTPRD*
Likely pathogenic	Abnormality of earlobe, Abnormality of mouth shape, Generalized hypotonia, Global developmental delay, Wide nasal bridge	*GLDC*	*UHRF2*
Pathogenic<br>Full	Cognitive impairment	*KANK1*	*DMRT1*

Likely pathogenic	Abnormality of the vasculature, Almond-shaped palpebral fissure, Delayed speech and language development, Epicanthus, Frontal bossing, Hypertelorism, Macrocephaly, Pulmonic stenosis, Wide nasal bridge	*MPDZ*<br>*NFIB*	*NFIB*
	Intellectual disability	*DOCK8*<br>*KANK1*<br>*SMARCA2*	*DMRT1*<br>*SMARCA2*
Likely pathogenic<br>Partial	Developmental regression, Trigonocephaly	*TYRP1*<br>*MPDZ*<br>*NFIB*	*NFIB*
	D+G163OCK8	*TYRP1*<br>*MPDZ*<br>*NFIB*<br>*FREM1*	*NFIB*
	Bilateral conductive hearing impairment, Delayed speech and language development, Intellectual disability, Mild conductive hearing impairment	*TYRP1*<br>*MPDZ*<br>*NFIB*<br>*FREM1*	*NFIB*<br>*PSIP1*
Likely pathogenic<br>Uncertain	Cleft palate, Premature birth	*DOCK8*<br>*KANK1*<br>*SMARCA2*<br>*VLDLR*<br>*KCNV2*<br>*GLIS3*	*DMRT1*<br>*SMARCA2*<br>*RFX3*

	Intellectual disability, Obesity	*SMARCA2*<br>*VLDLR*<br>*KCNV2*<br>*GLIS3*<br>*SLC1A1*<br>*JAK2*<br>*RIC1*<br>*GLDC*	*DMRT1*<br>*SMARCA2*<br>*RFX3*<br>*CDC37L1*<br>*JAK2*<br>*KIAA2026*<br>*UHRF2*
	Intrauterine growth retardation, Neurodevelopmental delay, Poor eye contact, Poor motor coordination, Precocious puberty	*DOCK8*<br>*KANK1*<br>*SMARCA2*<br>*VLDLR*<br>*KCNV2*<br>*GLIS3*<br>*SLC1A1*	*DMRT1*<br>*SMARCA2*<br>*RFX3*<br>*CDC37L1*
Likely pathogenic	Delayed speech and language development, Depressed nasal bridge, Epicanthus, Frontal bossing, Hyperextensibility of the finger joints, Hypertelorism, Pes planus, Shawl scrotum	*DOCK8*<br>*KANK1*<br>*SMARCA2*<br>*VLDLR*<br>*KCNV2*<br>*GLIS3*<br>*SLC1A1*<br>*JAK2*	*SMARCA2*<br>*RFX3*<br>*CDC37L1*<br>*JAK2*
Uncertain	Abnormal facial shape, Inappropriate behavior, Intellectual disability	*RIC1*<br>*GLDC*	*KIAA2026*<br>*UHRF2*<br>*PTPRD*
	Epileptic spasm, Intellectual disability, Intention tremor, Sleep disturbance, Strabismus	*DOCK8*<br>*KANK1*<br>*SMARCA2*<br>*VLDLR*<br>*KCNV2*<br>*GLIS3*<br>*SLC1A1*<br>*JAK2*<br>*RIC1*	*DMRT1*<br>*SMARCA2*<br>*RFX3*<br>*CDC37L1*

Likely pathogenic	Craniosynostosis, Hypopigmentation of hair, Hypopigmentation of the skin, Intellectual disability, Scoliosis	*TYRP1*<br><br>*MPDZ*<br>*NFIB*<br>*FREM1*	*PTPRD*<br><br>*NFIB*
Likely pathogenic	Abnormality of prenatal development or birth, Delayed speech and language development, Downslanted palpebral fissures, Downturned corners of mouth, Enlarged kidney, Finger clinodactyly, Macrotia, Mandibular prognathia, Vertebral fusion	*TYRP1*<br><br>*MPDZ*<br>*NFIB*<br>*FREM1*	*PTPRD*<br><br>*NFIB*
Likely pathogenic	Aplasia/Hypoplasia of the abdominal wall musculature, Clubbing, Craniosynostosis, Hypopigmentation of hair, Hypopigmentation of the skin, Intellectual disability, Microtia, Midface retrusion, Short neck, Wide intermamillary distance	*TYRP1*<br><br>*MPDZ*<br>*NFIB*<br>*FREM1*	*PTPRD*<br><br>*NFIB*
	Global developmental delay		

Pathogenic Full	Autistic behavior, Intellectual disability, mild, Upper limb muscle weakness	*DOCK8*<br>*KANK1*<br>*SMARCA2*<br>*VLDLR*<br>*KCNV2*<br>*GLIS3*<br>*SLC1A1*<br>*JAK2*<br>*RIC1*<br>*GLDC*	*DMRT1*<br>*SMARCA2*<br>*RFX3*<br>*CDC37L1*<br>*JAK2*<br>*KIAA2026*<br>*UHRF2*<br>*UHRF2*
	Abnormal heart morphology, Ambiguous genitalia, female	*DOCK8*<br>*KANK1*<br>*SMARCA2*<br>*VLDLR*<br>*KCNV2*<br>*GLIS3*<br>*SLC1A1*<br>*JAK2*<br>*RIC1*<br>*GLDC*	*DMRT1*<br>*SMARCA2*<br>*RFX3*<br>*CDC37L1*<br>*JAK2*<br>*KIAA2026*<br>*UHRF2*
	Frontal bossing, Hypertelorism, Hypotonia, Intellectual disability, Joint laxity	*TYRP1*<br>*MPDZ*<br>*NFIB*<br>*FREM1*	*PTPRD*<br>*NFIB*<br>*PSIP1*
	Autistic behavior, Blepharophimosis, Hypermetropia, Hypertelorism, Inappropriate sexual behavior, Poor motor coordination, Prominent forehead, Synophrys	*DOCK8*<br>*KANK1*<br>*SMARCA2*<br>*VLDLR*<br>*KCNV2*<br>*GLIS3*<br>*SLC1A1*<br>*JAK2*<br>*RIC1*<br>*GLDC*	*DMRT1*<br>*SMARCA2*<br>*RFX3*<br>*CDC37L1*<br>*JAK2*<br>*KIAA2026*<br>*UHRF2*<br>*UHRF2*<br>*PTPRD*
	Global developmental delay	*DOCK8*<br>*KANK1*<br>*SMARCA2*<br>*VLDLR*<br>*KCNV2*<br>*GLIS3*<br>*SLC1A1*	*DMRT1*<br>*SMARCA2*<br>*RFX3*<br>*CDC37L1*<br>*JAK2*<br>*KIAA2026*<br>*UHRF2*

		JAK2<br>*RIC1*<br>*GLDC*	*UHRF2*<br>*PTPRD*
	Abnormal nasolacrimal system morphology, Global developmental delay, Single umbilical artery, Trigonocephaly, Ventricular septal defect	*DOCK8*<br>*KANK1*<br>*SMARCA2*<br>*VLDLR*<br>*KCNV2*<br>*GLIS3*<br>*SLC1A1*<br>*JAK2*<br>*RIC1*<br>*GLDC*	*DMRT1*<br>*SMARCA2*<br>*RFX3*<br>*CDC37L1*<br>*JAK2*<br>*KIAA2026*<br>*UHRF2*<br>*UHRF2*<br>*PTPRD*
	Cryptorchidism, Delayed speech and language development, Generalized non-motor (absence) seizure, Intellectual disability, moderate, Obsessive-compulsive trait, Short nose, Thin vermilion border	*DOCK8*<br>*KANK1*<br>*SMARCA2*<br>*VLDLR*<br>*KCNV2*<br>*GLIS3*<br>*SLC1A1*<br>*JAK2*<br>*RIC1*<br>*GLDC*<br>*TYRP1*<br>*MPDZ*	*DMRT1*<br>*SMARCA2*<br>*RFX3*<br>*CDC37L1*<br>*JAK2*<br>*KIAA2026*<br>*UHRF2*<br>*UHRF2*<br>*PTPRD*
Pathogenic<br>Full	Anxiety, Hypertelorism, Long fingers, Protruding ear, Severe global developmental delay	*DOCK8*<br>*KANK1*<br>*SMARCA2*<br>*VLDLR*<br>*KCNV2*<br>*GLIS3*<br>*SLC1A1*<br>*JAK2*<br>*RIC1*<br>*GLDC*	*DMRT1*<br>*SMARCA2*<br>*RFX3*<br>*CDC37L1*<br>*JAK2*<br>*KIAA2026*<br>*UHRF2*<br>*UHRF2*<br>*PTPRD*

Pathogenic<br><br>Full	Abnormality of the pinna, Facial asymmetry, Intellectual disability, moderate, Knee flexion contracture, Lumbar hyperlordosis, Mild short stature, Proximal placement of thumb, Schizophrenia, Single transverse palmar crease, Small hand, Tapered finger	*DOCK8*<br><br>*KANK1*<br>*SMARCA2*<br>*VLDLR*<br>*KCNV2*<br>*GLIS3*<br>*SLC1A1*<br>*JAK2*<br>*RIC1*<br>*GLDC*	*DMRT1*<br><br>*SMARCA2*<br>*RFX3*<br>*CDC37L1*<br>*JAK2*<br>*KIAA2026*<br>*UHRF2*<br>*UHRF2*<br>*PTPRD*
Pathogenic<br><br>Full	Conductive hearing impairment, Gastroesophageal reflux, Global developmental delay, Overfolded helix, Sensorineural hearing impairment, Thin upper lip vermilion, Upslanted palpebral fissure	*DOCK8*<br><br>*KANK1*<br>*SMARCA2*<br>*VLDLR*<br>*KCNV2*<br>*GLIS3*<br>*SLC1A1*<br>*JAK2*<br>*RIC1*<br>*GLDC*	*DMRT1*<br><br>*SMARCA2*<br>*RFX3*<br>*CDC37L1*<br>*JAK2*<br>*KIAA2026*<br>*UHRF2*<br>*UHRF2*<br>*PTPRD*
	Diabetes mellitus, Hypospadias, Intellectual disability, Ureteral atresia	*DOCK8*<br>*KANK1*<br>*SMARCA2*<br>*VLDLR*<br>*KCNV2*<br>*GLIS3*<br>*SLC1A1*<br>*JAK2*<br>*RIC1*<br>*GLDC*<br>*TYRP1*<br>*MPDZ*<br>*NFIB*	*DMRT1*<br>*SMARCA2*<br>*RFX3*<br>*CDC37L1*<br>*JAK2*<br>*KIAA2026*<br>*UHRF2*<br>*UHRF2*<br>*PTPRD*<br>*NFIB*<br>*PSIP1*<br>*BNC2*

		FREM1	
	Abnormality of the musculature, Intellectual disability	*DOCK8*<br>*KANK1*<br>*SMARCA2*<br>*VLDLR*<br>*KCNV2*<br>*GLIS3*<br>*SLC1A1*<br>*JAK2*<br>*RIC1*<br>*GLDC*<br>*TYRP1*<br>*MPDZ*<br>*NFIB*	*DMRT1*<br>*SMARCA2*<br>*RFX3*<br>*CDC37L1*<br>*JAK2*<br>*KIAA2026*<br>*UHRF2*<br>*UHRF2*<br>*PTPRD*<br>*NFIB*<br>*PSIP1*<br>*BNC2*
	Choanal atresia, Hypotonia, Intellectual disability	*DOCK8*<br>*KANK1*<br>*SMARCA2*<br>*VLDLR*<br>*KCNV2*<br>*GLIS3*<br>*SLC1A1*<br>*JAK2*<br>*RIC1*<br>*GLDC*<br>*TYRP1*<br>*MPDZ*<br>*NFIB*<br>*FREM1*	*DMRT1*<br>*SMARCA2*<br>*RFX3*<br>*CDC37L1*<br>*JAK2*<br>*KIAA2026*<br>*UHRF2*<br>*UHRF2*<br>*PTPRD*<br>*NFIB*<br>*PSIP1*<br>*BNC2*
Pathogenic<br>Full	Trigonocephaly	*DOCK8*<br>*KANK1*<br>*SMARCA2*<br>*VLDLR*<br>*KCNV2*<br>*GLIS3*<br>*SLC1A1*<br>*JAK2*<br>*RIC1*<br>*GLDC*<br>*TYRP1*<br>*MPDZ*	*DMRT1*<br>*SMARCA2*<br>*RFX3*<br>*CDC37L1*<br>*JAK2*<br>*KIAA2026*<br>*UHRF2*<br>*UHRF2*<br>*PTPRD*<br>*NFIB*
