# Supplementary material for: Inherited deletion of 9p22.3‐p24.3 and duplication of 18p11.31‐p11.32 associated with neurodevelopmental delay: Phenotypic matching of involved genes
Source: J Cell Mol Med. 2023 Jan 24;27(4):496–505. doi: 10.1111/jcmm.17662 (PMC9930415; doi:10.1111/jcmm.17662)
Supplement: Supplementary file 4 — Table S3. [file JCMM-27-496-s004.pdf]

Cytoband	Gene	OMIM	GnomAD pLI	%HI
	FOXD4	601092	0	
	CBWD1	611078	0	69.8
	DOCK8	611432	0	35.68
	KANK1	607704	0	49.73
	DMRT1	602424	0.74	18.6
	DMRT3	614754	0	58.28
	DMRT2	604935	0.01	53.86

9p24.3	*SMARCA2*	600014	1	1.6
9p24.2	*VLDLR*	192977	0	13.4

	KCNV2	607604	0	44.67
	RFX3	601337	1	7.5
	GLIS3	610192	0	2.59
	SLC1A1	133550	0	31.17
9p24.1	*SPATA6L*		0	74.04
	PLPP6	611666	0	
	AK3	609290	0	40.45
	CDC37L1	610346	0.97	16.72
	RCL1	611405	0.06	16.34
	JAK2	147796	0.65	0.82
	INSL6	606414	0	88
	INSL4	600910	0	98.3
	RLN2	179740	0	99.82
	RLN1	179730	0.01	99.45
	PLGRKT	618444	0	43.19
	CD274	605402	0.02	74.86
	PDCD1LG2	605723	0	84.51
	RIC1	610354	0	
	ERMP1	611156	0	47.36
	KIAA2026		0.66	36.7
	MLANA	605513	0	65.87
	RANBP6		0	36.47
	IL33	608678	0	91.78
	TPD52L3	617567	0.28	84.98
	UHRF2	615211	1	18.75
	GLDC	238300	0	30.92
	KDM4C	605469	0	37.27
	DMAC1	617261	0.01	
	PTPRD	601598	1	0.14

9p23	*TYRP1*	115501	0	21.83
	LURAP1L	616130	0.03	36.91
	MPDZ	603785	0	33.44
	NFIB		1	0.38
9p22.3	*ZDHHC21*	614605	0.03	9.4
	CER1	603777	0	65.98
	FREM1	608944	0	26.8
	TTC39B	613574	0	44.57
	TUBB8B		0	
	USP14	607274	0.91	4.56
	THOC1	606930	1	12.41
	COLEC12		0..91	23.25
	CETN1	603187	0.22	64.7
	CLUL1	616990	0	73.93

18p11.32	*TYMSOS*		0.16	
	TYMS	188350	0.72	4.62
	ENOSF1	607427	0	50.06
	YES1	164880	0	5.23
	ADCYAP1	102980	0.52	43.32
	METTL4		0	61.87
	NDC80	607272	0	31.04
	SMCHD1	614982	1	27.68
18p11.31	*EMILIN2*	608928	0	73.29
	LPIN2	605519	0	38..45
	MYOM1	603508	0	40.63
	MYL12A		0	29.39
	MYL12B	609211	0.11	26.69
	TGIF1	602630	0.92	30.38

G2P	Disease/s
Biallelic	Hyper-IgE recurrent infection syndrome (AR)
Imprinted (possible)	Cerebral palsy, spastic quadriplegic, 2 (U)

Monoallelic	Blepharophimosis-impaired intellectual development syndrome (AD)<br>Nicolaides-Baraitser syndrome (AD)
Biallelic	Cerebellar hypoplasia and mental retardation with or without quadrupedal locomotion 1 (AR)

Biallelic	Retinal cone dystrophy 3B (AR)
Biallelic	Hydrocephalus, congenital, 2, with or without brain or eye anomalies (AR)
	Dicarboxylic aminoaciduria (AR)<br>Schizophrenia susceptibility 18 (U)
	Budd-Chiari Syndrome; BDCHS (U)<br>Erythrocytosis, Familial, 1; ECYT1 (U)<br>Leukemia, Acute Myeloid; AML (U)<br>Myelofibrosis (U)<br>Polycythemia vera, somatic (U)<br>Thrombocythemia 3 (AD, S)
Biallelic	CATIFA syndrome (AR)
Biallelic	Glycine encephalopathy (AR)

Biallelic	Albinism, oculocutaneous, type III (AR)<br>Skin/hair/eye pigmentation, variation in, 11 (Melanesian blond hair) (U)
Biallelic	Hydrocephalus, congenital, 2, with or without brain or eye anomalies (AR)
Monoallelic<br>(possible)	Macrocephaly, acquired, with impaired intellectual development (AD)
Biallelic	Bifid nose with or without anorectal and renal anomalies (U)<br>Manitoba oculotrichoanal syndrome (AR)<br>Trigonocephaly 2 (AD)

Monoallelic	Bosma arhinia microphthalmia syndrome (AR)<br><br>Fascioscapulohumeral muscular dystrophy 2, digenic (Digenic dominant)
	Majeed syndrome (U)
	Hypertrophic Cardiomyopathy
Monoallelic	Holoprosencephaly 4 (AD)

PhenogramViz matched phenotype/s
Intellectual disability, neurodevelopmental delay, global developmental delay, cognitive impairment, delayed speech and language development
Corpus callosum atrophy, cognitive impairment, intellectual disability, muscular hypotonia
Abnormality of the penis
Abnormality of the penis

Intellectual disability, neurodevelopmental delay, global developmental delay, cognitive impairment, delayed speech and language development, motor delay, low posterior hairline, thick eyebrows, highly arched eyebrow, thin upper vermilion, long philtrum, open mouth, anteverted nostrils, wide nasal bridge, wide intermamillary distance, tapered finger, pes planus
Intellectual disability, neurodevelopmental delay, global developmental delay, cognitive impairment, delayed speech and language development, Corpus callosum atrophy, motor delay, pes planus

Ventricular septal defect
Intellectual disability, cognitive impairment, Corpus callosum atrophy
Corpus callosum atrophy, low posterior hairline, thick eyebrows, highly arched eyebrow, anteverted nostrils, wide nasal bridge,<br>Elevated transferrin saturation
Abnormality of the penis
Abnormality of the penis
Corpus callosum atrophy
Low posterior hairline, highly arched eyebrow, thick eyebrow
Intellectual disability, cognitive impairment, corpus callosum atrophy, muscular hypotonia

Corpus callosum atrophy, low posterior hairline, highly arched eyebrow, thick eyebrow
Intellectual disability, cognitive impairment,
Corpus callosum atrophy, highly arched eyebrow, thick eyebrow
Low posterior hairline, highly arched eyebrow, thick eyebrow
Trigonocephaly, low posterior hairline, Highly arched eyebrow, thick eyebrow, broad nasal beige, hypertelorism, anteverted nostrils, long philtrum, Tapered finger
Corpus callosum atrophy, pes planus, Abnormality of the penis

Trigonocephaly
Trigonocephaly, corpus callosum atrophy
Low posterior hairline, highly arched eyebrow, thick eyebrow, pes planus
Trigonocephaly, corpus callosum atrophy, thick eyebrows, highly arched eyebrow, thin upper vermilion, long philtrum, anteverted nostrils, wide nasal bridge, hypertelorism
